# Efficient Maximum Likelihood Estimation of Kinetic Rate Constants from Macroscopic Currents

**DOI:** 10.1371/journal.pone.0029731

**Published:** 2011-12-29

**Authors:** Andrey R. Stepanyuk, Anya L. Borisyuk, Pavel V. Belan

**Affiliations:** 1 Bogomoletz Institute of Physiology, Kiev, Ukraine; 2 State Key Laboratory of Molecular and Cellular Biology, Kiev, Ukraine; University of California, Berkeley, United States of America

## Abstract

A new method is described that accurately estimates kinetic constants, conductance and number of ion channels from macroscopic currents. The method uses both the time course and the strength of correlations between different time points of macroscopic currents and utilizes the property of semiseparability of covariance matrix for computationally efficient estimation of current likelihood and its gradient. The number of calculation steps scales linearly with the number of channel states as opposed to the cubic dependence in a previously described method. Together with the likelihood gradient evaluation, which is almost independent of the number of model parameters, the new approach allows evaluation of kinetic models with very complex topologies. We demonstrate applicability of the method to analysis of synaptic currents by estimating accurately rate constants of a 7-state model used to simulate GABAergic macroscopic currents.

## Introduction

Markov models are a powerful tool for a statistical description of voltage- and ligand-gated ion channels [1], [2]. Operating with a transition matrix, they represent the whole available information about the kinetic properties of a channel in a compact form, allowing simulation of ion channel behavior [3], [4], comparison of different channel subtypes [5], [6], investigation of its modulated states [7], [8] and its interactions with pharmacological agents [9], [10]. States and transitions of kinetic model map onto conformational states and transitions of ion channel proteins [11], [12]. Thus, ion channel kinetic models can be useful tools for investigating ion channel structure and function at the molecular level [11], [13].

The standard methods of estimation of kinetic rates are based on the statistical analysis of single-channel patch-clamp recordings [1], [14]–[19]. But it is also possible to use for this purpose the macroscopic currents, i.e. currents generated by an ensemble of identical ion channels [20]–[22]. Not only has this approach an advantage of more simple and fast recording procedure, but it also makes possible to maintain the natural biochemical environment of ion channels during the recordings. Besides, the macroscopic current approach becomes especially useful and, in most cases the only applicable approach, when synaptic channel properties are evaluated.

Several methods of statistical estimation of kinetic rates from macroscopic currents have been recently described for kinetic models with a known topology [20]–[23]. However, methods, which utilize Hidden Markov Models [17], [22], [24] are computationally expensive. The number of operations necessary to estimate model parameters increases exponentially with a model complexity and the number of channels contributing to the macroscopic currents [22]. So these methods are hardly applicable to the majority of experimental data.

Other methods are based on the approximation of the macroscopic current by a Gaussian process. Some of them do not make use of the local time correlations, which is contained in the macroscopic current fluctuations [21]. It substantially reduces the number of necessary operations, although the accuracy of these methods is compromised as a result [22]. On the other hand, regarding the local time correlations using covariance fitting scales the amount of calculations as the square of the number of points in the macroscopic current, making this method limited with regards to the number of points it can use [20]. The problems mentioned above are overcome in a recursive algorithm, which utilizes Kalman filter for the maximum likelihood estimation of kinetic parameters [22]. However, the number of operations required in this method increases as the third power of the number of states in a model that can substantially slow down the calculation in the case of complex channel models.

In this work we have developed an alternative approach to the maximum likelihood estimation (MLE) of the channel kinetic model parameters. We have started from the expression of the macroscopic current likelihood as a function of kinetic model [18], [20]. Then we have noticed that the covariance matrix of macroscopic currents is quasiseparable. Efficient linear algebra algorithms for such matrices [25]–[27] provided a method for the exact likelihood logarithm (log-likelihood) calculation that takes into account statistics of local time correlations and scales approximately linearly with the number of states in a kinetic model. Moreover, using semiseparable representation of covariance we have substantially accelerated the log-likelihood gradient calculation due to a new approach having a weak dependence of the amount of required operations on the number of model parameters.

## Materials and Methods

### The Model of a Macroscopic Current

In this work we consider an ensemble of independent and identical ion channels. Behavior of each channel is described by the Markov process and 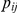 is a probability of the channel transition from the state 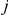 to the state 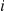 during the time interval 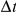. Then, 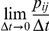 gives a rate constant of the transition 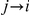. A macroscopic current elicited in response to an external stimulation of the channels is assumed to be a sum of single-channel currents and noise. The external stimulation is modeled as an instantaneous change in those transition probabilities, which depend on the neurotransmitter concentration (if ligand-gated channels are under study) or on the membrane potential (in the case of voltage-gated channels).

In this study we consider two types of stimulation protocols. In the first type, an external stimulation is a single step change of the concentration or the voltage and there is no additional stimulations during the current recording. This is referred to as a simple protocol. The second type of stimulation protocols, referred to as a complex protocol, includes series of steps of various amplitudes and durations during the current recording.

The macroscopic current is sampled at discrete time intervals. The model parameters are: rate constants, 
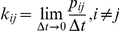
, currents, 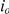, flowing through the channel being in each of its conducting states 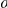, and the number of channels in the ensemble, 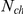. These parameters form the parameter vector, 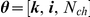, where 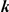 is the set of unknown rate constants. The kinetic model topology, i.e. the number of conformational states of the channel, the set of allowed transitions between them and the set of conducting (open) states are assumed to be known.

### Asymptotic Log-likelihood

To describe a state of a given ion channel at each time point, let us introduce a random vector 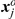, so that 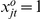 if the channel 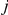 is in a conducting state 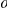 at time 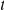, and 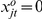 otherwise. Since the macroscopic current is assumed to be the sum of single-channel currents, it is described by the sum of such vectors, 
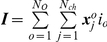
. Vectors 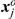 are statistically independent and identically distributed. In the limiting case of large number of channels, 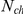, according to the multidimensional central limit theorem, the distribution of the sum of vectors 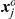 converges to the multivariate Gaussian distribution. Then the likelihood of macroscopic currents, i.e. the probability density of a particular set of 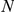 macroscopic current traces, 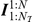, each consisting of 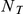 points, is given by [18], [20], [21]

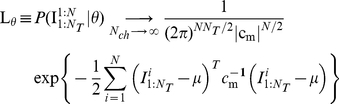
(1)Here 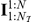 is 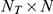 matrix composed of the macroscopic current traces, 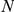 is the number of traces and 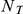 is the number of points in each macroscopic current, 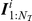; 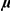, a vector of 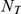 dimension with elements 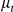 and 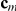, an 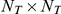 matrix with elements 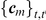 denote mean and covariance of the current, respectively, and they both are the functions of 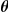.

The mean and covariance of macroscopic current in the case of simple stimulation protocol follow equations [18] (see A0 in Text S1 for derivation):
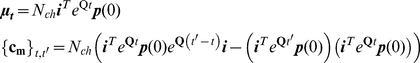
(2)Here 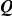 is a rate matrix [18], [20] and 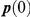 is an initial state vector. This vector can be expressed as a function of kinetic model provided that the concentration/voltage stimulation applied to the channels is known during sufficiently long time 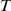 preceding current registration:
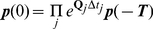
(3)In general, likelihood 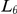 tends to Gaussian function of model parameters provided the number of traces is large enough and they are statistically independent.

The estimate of the most likely parameter set of the model, 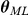, is now given by
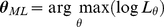
(4)The diagonal elements of the inverse Hessian matrix of 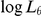, taken at the point 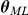, approximate the variance of parameter estimates [22]

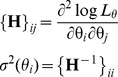
(5)One can see that, in general case, the calculation of the log-likelihood (Eq. 1) requires 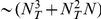 elementary operations (i.e. operations of the form 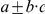 [27]). However, the log-likelihood can be estimated more efficiently using the fact that 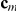 has specific semiseparable structure expressed by Eq. 2.

### Fast Calculation of Log-likelihood

To approach the problem of the computationally efficient estimation of the kinetic model, let initially consider a linear dynamical system of the form:
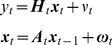
(6)Where 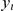 is observable variable, 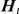 - 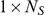 vector, 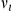 is a stationary Gaussian noise variable with a known autocorrelation function, 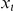 is a hidden 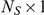 variable, 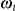
∼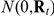 is a random 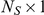 variable.

Then it is easy to show that the covariance of the observable variable has the form:
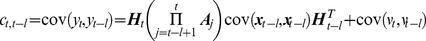
(7)The likelihood of the set of model parameters, 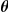, is a probability to obtain the vector of observable variable, 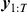, given this set of parameters. In the case of the model expressed by Eq. 6, this probability has Gaussian distribution and the log-likelihood of 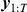 is:
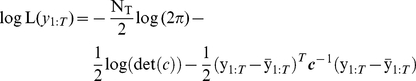
(8)Efficient estimation of the log-likelihood function can be done using Cholesky factorization of 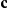.
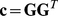
(9)where 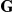 is a lower triangular matrix: 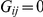, 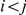. It is well known that matrix 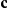 is quasiseparable [28], [29]. This remarkable property of covariance is clearly seen from the Eq.7. For quasiseparable matrices there are several fast algorithms for computing Cholesky factorization ([25]–[27] and A1 in Text S1).

The use of factorization allows us to rewrite the log-likelihood (Eq. 2) in the form:
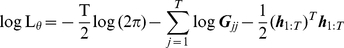
(10)The matrix 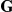 is also quasiseparable (see A1 in Text S1), and vector 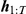 is the solution of the system of equations (A1 in Text S1).
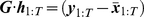
(11)One iteration of the algorithm can be now summarized as follows (see A1 and A3 in Text S1 for details):

For 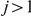:

Update statistics of unconditioned process (follows from Eq. 6):
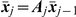


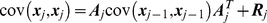
(12)

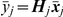



Update components of quasiseparable representation of 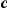 (Eq. 7):
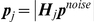


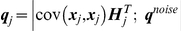


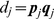
(13)

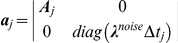



Update Cholesky decomposition of covariance (Eq.9):
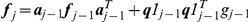


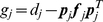
(14)

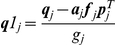



Measurement (Eq. 11):
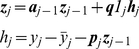
(15)


Update of the likelihood (Eq. 12):
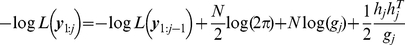
(16)


For 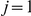:
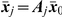


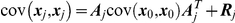


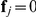


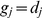
(17)

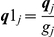


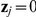


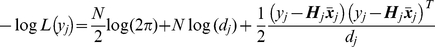
This recursive implementation of the algorithm can be computationally more efficient than Kalman filter when 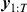 contains 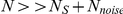 independent threads of data, since all calculations described in Eqs. 15–16 require an order of 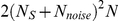 elementary operations and no other calculation depends on 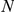. Additional improvement in performance can be achieved if the matrix 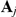 does not change every time step when the data is sampled. In this case using eigendecomposition it can be represented in the form: 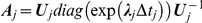, where 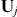 is a matrix composed of eigenvectors and 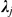 is a vector of eigenvalues of the matrix 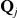 such that 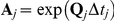.

Then Eq. 13 should be transformed as follows:
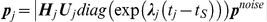


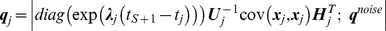
(18)

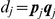


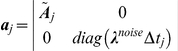
where 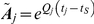 if 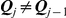 and 
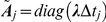
 otherwise; 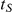 is the time moment when 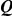 was changed last time. After this transformation one iteration of the algorithm requires approximately 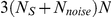 operations if 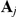 was not changed during this iteration.

In a context of macroscopic currents, 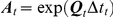, where 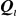 is a rate matrix, 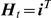, 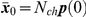 and 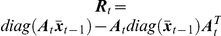 (see, for example, Eq. 53 in [20]).

Using Eqs.12 it can be shown that for the macroscopic currents 
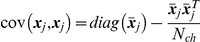


If the rate matrix changes several times during the time interval 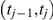, then 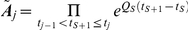.

### Efficient Estimation of the Log-likelihood Gradient

To search for the maximum of 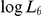 using a convex optimization it is necessary to estimate its gradient, 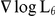: 
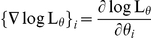
.

The gradient can be numerically estimated using finite difference method:
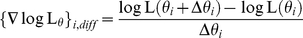
(19)The calculation of the gradient using this direct approach requires 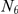 times more elementary operations then required for the calculation of the log-likelihood itself, where 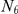 denotes the size of the parameter vector 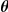. The more efficient approach is based on the fact that matrices required for the calculation of the log-likelihood gradient in the case of the simple protocol are semiseparable or quasiseparable. Indeed, differentiating log-likelihood (Eq. 1) with respect to 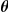 and using Eq.18, we have:
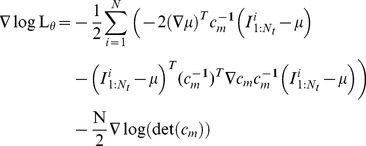
(20)where
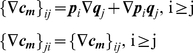
(21)Therefore, the matrix 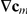 is symmetric semiseparable. As it is shown in Text S1, A2, in order to compute 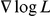 using Eq. 20, in addition to Cholesky factorization of matrices 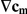 and 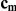 and solving the system of equations with coefficient matrices being lower and upper triangular, the following operations are necessary: multiplication of semiseparable matrix by vector, semiseparable matrix inversion and two operations of the form 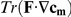. Here 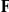 is either an 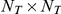 semiseparable matrix with component matrices of the size 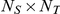 and 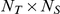 (similar to that from Eqs. 18) or 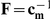 is a quasiseparable matrix (see A2 in Text S1).

The detailed algorithm of the gradient estimation is described in Text S1, A2.

It appears that the complexity of calculation of 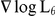 is independent of the length of the vector 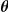. For 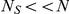 the calculation of gradient requires 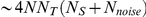 operations compared to 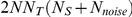 operations for log-likelihood computation (see A2 in Text S1).

### Noise Model

Noise observed in the patch-clamp recordings can be considered as the sum of two uncorrelated processes: background noise and open-channel excess noise [30].

The background noise can be well approximated by a stationary Gaussian process [17], [19]. The power spectral density of the experimental background noise typically has the form:
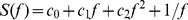
(22)


Its components arise mainly from shot and thermal noises imposed onto the patch, electrode and hardware capacitance and, sometimes, it has an additional 1/f component associated with the noise of neuronal membrane [30], [31].The covariance matrix of stationary background noise is Toeplitz and can be well approximated by a low order semiseparable matrix.

In this work we assume that background noise is Gaussian and model it as the sum of 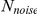 = 1÷4 first-order autoregressive (AR) processes [17], [19]:
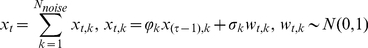
(23)


The spectral density of the resulting process is a sum of Lorentzians and it can well approximate Eq. 22 [17], [19]. The covariance matrix of 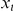 has a form
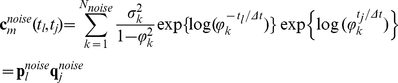
(24)because the autocorrelation of AR(1) process is a decaying exponential.

Since the macroscopic current covariance is the sum 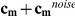, it is quasiseparable with components, expressed by Eq.13 or Eq.18.

The background noise statistics (i.e. parameters 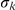 and 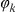) can be obtained from parts of recordings, where signal is not present, using the maximum likelihood estimation algorithm (Eqs.12–18). To obtain initial estimates of 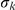 and 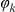, used as a starting point for the maximization of the noise log-likelihood, a statistical estimate of noise autocorrelation was fitted with the sum of several exponentials (Eq. 24).

The open-channel noise is usually associated with shot noise in the single-channel current as well as with conformational fluctuations of the channel protein [32], [33]. The excess noise of the closed state is zero and a standard deviation for the open state is typically about 3% of the single channel current [32].

Assuming correlated open-channel noise, one must have an *a priori* knowledge about the noise model and statistics. In some cases this information can be obtained from single-channel experiments with a particular type of the ion channel [19], [32], [34]. Neglecting the correlations of noise with macroscopic current [30], the covariance matrix of noise can be added to the covariance matrix of the current. Alternatively, the excess noise can be considered as arising from open channel transitions between several additional subconductance levels or it can be the consequence of the fast openings and closings of the channel [35]. In this case the estimation of the noise model can be treated as a specific case of the model topology selection problem (see Discussion).

In this work, we used two models of excess noise: 1) In the first model, for simplicity, open-channel noise was assumed to be white [32]. Thus, the term
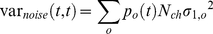
(25)was added to the diagonal of the covariance matrix of the macroscopic current. Here 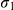 is the standard deviation of the open-channel noise for a single channel, 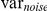 is the variance of the open-channel noise for the macroscopic current.

2) In the second model each open state was split into two states with single-channel currents 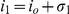 and 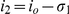. The rate of transitions between these states was 100 ms^−1^. The parameters of noise models (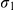 for the first model and 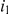, 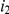 for the second one) were then estimated together with the kinetic constants.

Summing up, the algorithm, we have introduced, is quite general as it can be used for the estimation of ion channel kinetic models from the macroscopic currents under Gaussian colored background and open-channel excess noises.

### Log-likelihood Global Maximum Search

Finally, to obtain the required model parameters (rate constants, conductances and the number of channels) from a set of macroscopic currents, we search for the log-likelihood global maximum. In order to do this, we minimize negative log-likelihood using graduated optimization. Initial estimates of each parameter are randomly and uniformly chosen from the logarithmic scale interval, 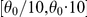, where 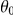 is a vector composed of the true parameter values, i.e. of values utilized by the macroscopic current generator (see below).

The whole minimization procedure is divided into sequential minimization steps. On the first step negative log-likelihood of the first 5 currents regularly sampled at 50 points each is minimized, and the estimated parameters are taken as initial parameters for the next minimization step. The procedure is repeated for each consequent step in the following manner: for the second step we sample each of the 5 currents at 100 points, and for the third step – at 200 points. Next, we sample currents at 200 points but consequently increase the number of currents on each step, taking N = 7, 10, 14, 18, 25, 30, 40, 50, 66, 85, 100, 125, 150, 200, 250, 330, 400 and 500 currents for minimization. On the final steps we minimize negative log-likelihood of the whole set of 500 currents and sample them, on each step, at 200, 400, 800, 1600 and finally at 2500 points. When the dependence of the algorithm performance on the number of currents is tested (see Results), we stop to increase the number of currents, taken for minimization, on a desired value of N, and then increase the number of points from 200 to 2500 in the abovementioned manner.

To test the convergence of the algorithm, we have minimized the negative log-likelihood of a particular set of 200 currents using 40 different randomly chosen initial sets of parameters. The algorithm failed to find a global minimum in less than 10% of all cases. The values of log-likelihood in its maximum differed between samples by no more than 10^−6^ for all cases when global minimum was found.

Thus, for all calculations in this work we rerun minimization 3 times, each time starting from the different initial parameter set and then chose those of the parameter estimates which had the best log-likelihood. In doing so we obtained negative log-likelihood global minimum with the probability 99.9%.

Minimization was done using sequential quadratic programming (SQP) method embedded in MATLAB Optimization toolbox function *fmincon*. During the search, all parameters were bounded within the interval 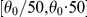. The linear/nonlinear equality or inequality constraints on the parameters, described elsewhere [21] can be also easily imposed, because they are inherent to the *fmincon* function, designed to find a minimum of nonlinear constrained multivariable function.

### Macroscopic Current Generator

**Figure 1:**
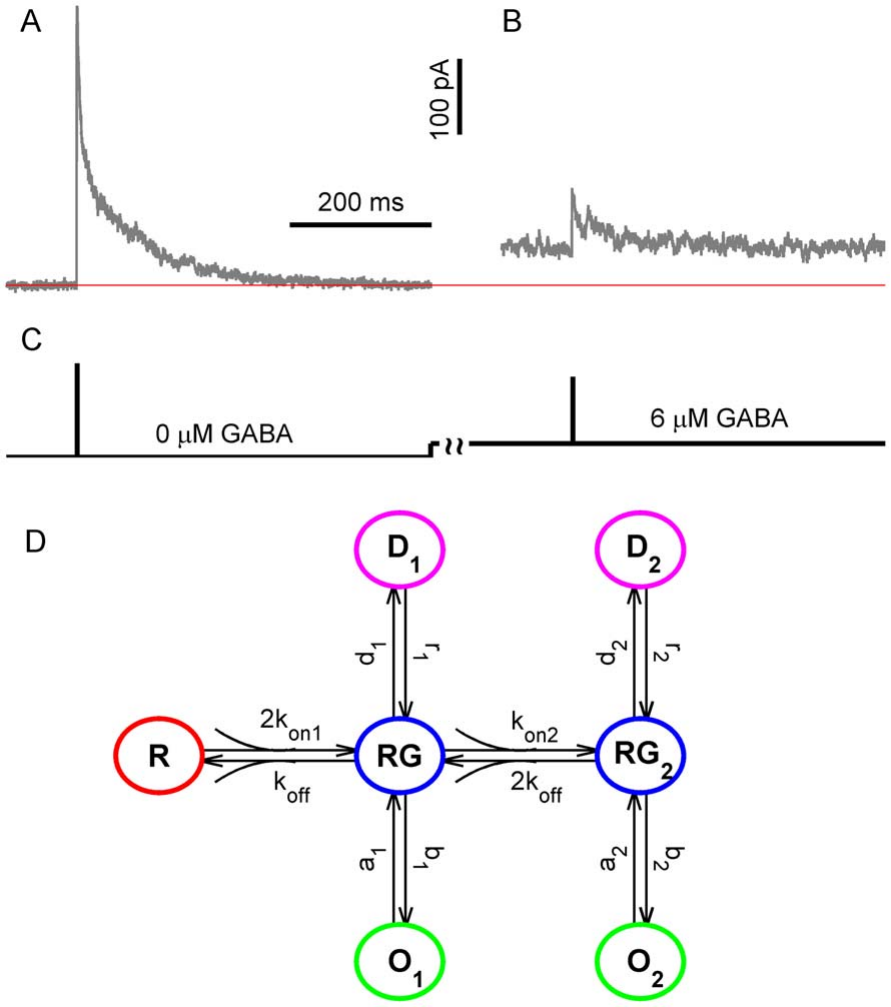
GABA_A_ receptor model, stimulation protocols and simulated currents. Macroscopic currents were simulated by Monte-Carlo method using a standard kinetic scheme of synaptic GABA_A_ receptor [36]. (A) Simulated currents produced by a brief (0.2 ms) application of saturating GABA concentration to 500 unliganded receptors. A thin red horizontal line corresponds to a zero current. White noise with the standard deviation of 3 pA was added to the initially generated currents. (B) Simulated currents evoked by saturating GABA application to receptors that were preincubated with 6 µM GABA. (C) Protocol of GABA applications for panels A and B. (D) The kinetic model used in simulations of macroscopic GABA_A_ receptors. The model consists of single and double-bound closed states RG and RG_2_, that are linked to corresponding open and desensitized states O_1_, D_1_ and O_2_, D_2_, respectively. Both single- and double-liganded open states are thought to have the same conductance of 1 pA. Rate constants (adapted from ref. [36]) were as follows: 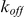 = 0.13, 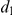 = 0.14, 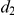 = 1.5, 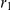 = 0.02, 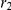 = 0.12, 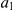 = 1.5, 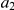 = 1, 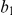 = 0.15, 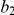 = 8 (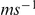); 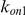 = 4, 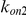 = 8 (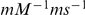).

We tested the performance of the algorithm with a set of macroscopic currents generated by Monte-Carlo simulation. In the most part of this work we did that for GABA_A_ currents using a kinetic scheme of GABA_A_ receptor [36] which is shown in Fig. 1. Kinetic rates were adapted from [36] and were as follows: 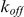 = 0.13 ms^−1^, 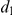 = 0.14 ms^−1^, 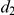 = 1.5 ms^−1^, 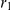 = 0.02 ms^−1^, 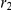 = 0.12 ms^−1^, 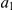 = 1.5 ms^−1^, 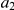 = 1 ms^−1^, 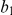 = 0.15 ms^−1^, 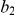 = 8 ms^−1^; 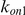 = 4 mM^−1^ ms^−1^, 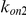 = 8 mM^−1^ ms^−1^; 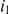 = 1 pA, 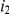 = 1 pA; 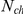 = 500; An example of the simulated current is given by Fig. 1 A, B.

Two different types of simple stimulation protocols (Fig. 1 C) were used to generate the macroscopic currents. According to the first protocol, referred to as brief stimulation, the receptors were stimulated by a brief (modeled as a 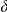-function) application of saturating GABA concentration. In this case all channels were considered to be in RG_2_ state right after a termination of GABA application. In the second protocol (referred to as brief stimulation with preincubation) receptors were persistently activated by GABA having a low (6 µM) constant concentration and the same brief application of saturating GABA concentration was done on top of this concentration (Fig. 1 B)

A total of 1000 currents were simulated using each of these two protocols. Sampling interval for each current was chosen to be 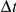 = 0.2 ms. The segments of simulated currents from 1 ms to 501 ms after the brief stimulation were taken for the consequent log-likelihood maximization.

For the most part of this work we have considered currents under white noise. In order to do this a random number taken from the normal distribution with zero mean and variance 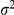 = 9pA^2^ was added to each generated current at each time point. In addition, we have conducted several computational experiments with more realistic noise model. Background noise was modeled as a sum of 4 AR(1) processes with parameters 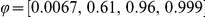 (when the sampling interval was 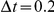 ms) and 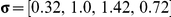, pA (obtained from the approximation of the whole-cell patch clamp background noise autocorrelation function by the sum of 4 exponentials, see Eqs. 23–24). The open-channel excess noise was modeled as white noise with standard deviation (per open channel) 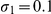 pA for each conducting state.

In order to directly validate that our approach may evaluate the fast opening rate constants within channel models obtained using a single channel analysis [37]–[40] we have also generated macroscopic GABA_A_ receptor currents based on recently published detailed model (Figure 7A in [41]). The model has been modified by increasing the opening and closing rate constants for one of the channel conducting states (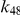 and 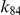) from 1.66 to 66.4 ms^−1^ and from 1.986 to 7.944 ms^−1^, respectively. The kinetic model parameters were as follows: 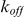 = 0.33 ms^−1^, 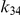 = 0.521 ms^−1^, 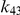 = 1.362 ms^−1^, 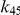 = 1.648 ms^−1^, 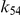
0.34 ms^−1^, 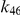 = 0.205 ms^−1^, 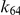 = 0.223 ms^−1^, 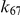 = 1.216 ms^−1^, 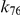 = 0.153 ms^−1^, 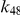 = 66.4 ms^−1^, 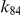 = 7.944 ms^−1^; 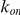 = 17 mM^−1^ ms^−1^; 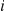 = 3 pA, 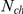 = 500. Five sets of 500 currents each were generated by Monte-Carlo method as described for the previous model. The macroscopic current was simulated using the 30-µs application of saturating GABA concentration on top of the respective constant GABA concentration (0, 2, 6, 15 and 5000 µM for each set, respectively). Each of 725 parameter searches utilized 5 sets of 50 currents that were randomly selected for each GABA concentration from the initially generated sets. Sampling interval was set at 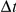 = 30 µs.

Realistic colored noise was added to the currents. Parameters of noise model were obtained from whole-cell recordings of cultured hippocampal neurons using a sampling interval of 30 µs. The recordings were filtered and with 30 kHz analog 3-pole Bessel filter. The root mean square of noise (12.4 pA) and noise parameters 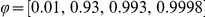 and 
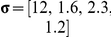
, pA were obtained from the approximation of the whole-cell patch clamp background noise autocorrelation function by the sum of 4 exponentials. A single-channel current amplitude was set at 3 pA, which is comparable with GABA_A_ channel current recorded at 100–120 mV driving force.

### Estimation of Errors

The errors of the maximum likelihood approximation of the kinetic rates, described in this work, were estimated with a bootstrap analysis. To do that 20 to 40 bootstrap samples were generated for each stimulation protocol sampling with replacement from initially generated set of 1000 traces.

For each bootstrap sample we rerun minimization 3 times, each time starting from different initial parameters. The estimated model parameters, 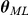, were obtained from the trial that resulted in the best log-likelihood and was considered to be a global maximum.

The accuracy of estimated model parameters was evaluated as a deviation of these parameters (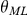) from those (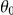) used for the generation of the currents (i.e. 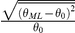).

The algorithm was implemented in MATLAB (see A4 in Text S1). Source codes are freely available at: http://code.google.com/p/multi-channel-data-analysis/.

## Results

### Estimation of Model Parameters Using a New MLE Method

We tested the performance of our algorithm on simulated GABA_A_ receptor macroscopic currents (Macroscopic Current Generator in Methods). A standard model of this ligand-gated channel was chosen for the simulations [42] and two types of simple stimulation protocols were implemented during the simulations: (i) a brief application of saturating GABA concentration (brief stimulation) to unliganded receptors, and (ii) the same application of saturating GABA concentration applied to the receptors, preincubated with a low (6 µM) constant concentration of GABA (brief stimulation with preincubation, Methods and Fig. 1 C). The first protocol resembles synaptic GABA release in certain type of synaptic connections [34], [43]–[48] while the second one enables estimating the time constants of GABA binding (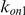, 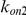), which otherwise could be hardly derived from the macroscopic traces obtained within the first protocol.

A set of 1000 macroscopic currents was generated for each protocol and examples of such currents are shown in Fig. 1 A and B, respectively. The currents evoked by a brief stimulation had the mean amplitude of 370 pA and a decay well approximated by a sum of two exponentials with amplitudes of 159 pA and 149 pA and decay times of 5.2 and 89 ms, respectively. The currents resemble postsynaptic currents routinely recorded in cortical GABAergic synapses [49]. Steady state currents generated in a response to a low level of constant GABA concentration (6 µM) had the mean amplitude of 50 pA with variance of 32 pA^2^. Channel state occupancies during these steady state currents were as follows: R – 18.3%, RG – 6.8%, RG_2_ – 1.3%, O_1_ – 0.7%, O_2_ -10%, D_1_ -47.4%, D_2_ – 15.6%. Thus, both single and double-bound states were reasonably represented in the generated currents, enabling estimation of 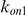, 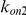 from current fluctuations. The mean amplitude of macroscopic currents generated using the brief stimulation with preincubation was 78 pA (Fig. 1B).

**Figure 2:**
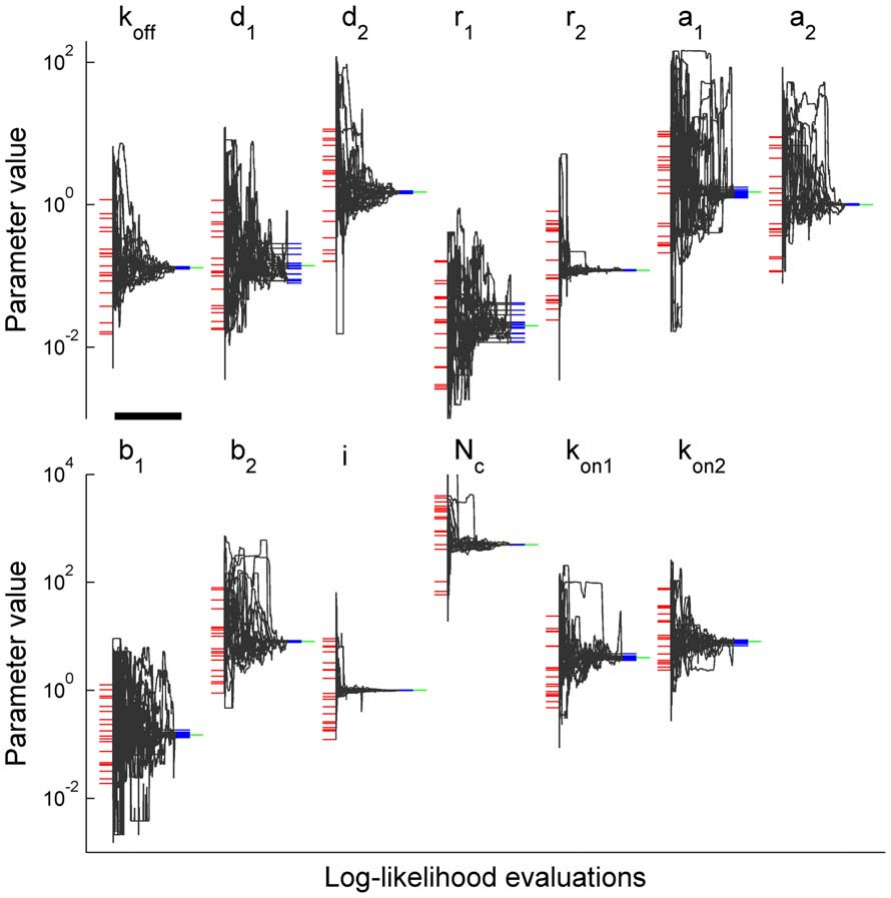
Convergence of the method for the case of combined dataset. Each of 20 performed parameter searches utilized two sets of simulated currents: 100 currents without and 100 currents with preincubation with 6 µM GABA, similar to that used in Fig. 1 A–C. Each black trace shows how the estimate of parameter indicated on the top evolved during maximization of the likelihood of a particular set of currents. Each point in the traces represents an iteration of the maximization algorithm. Initial values of each parameter (red dashes) were chosen randomly and uniformly in the logarithmic scale from the interval 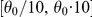, where 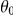 denotes their true values (green dashes). Blue dashes mark parameter values, to which the algorithm converged. The algorithm converged for all estimated parameters. A black horizontal bar corresponds to 2500 log-likelihood evaluations.

Using our algorithm the model parameters were estimated from a set of two groups of currents: 100 currents evoked by the brief stimulation and 100 currents evoked by the brief stimulation with preincubation. These two groups were combined for the analysis, and their log-likelihood was estimated as a sum of log-likelihoods of each group. Each group of 100 currents was randomly sampled with replacement from the corresponding initially generated set of 1000 currents. To estimate the algorithm accuracy, the parameter search was performed for 20 sets of 200 currents obtained in the above manner (see Log-likelihood Global Maximum Search in Methods for details). For each run, the initial parameter values (red lines in Fig. 2) were chosen randomly and uniformly in the logarithmic scale from the range 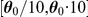, where 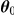 denotes a vector of true parameter values, i.e. parameters used for the macroscopic current generation.

**Figure 3:**
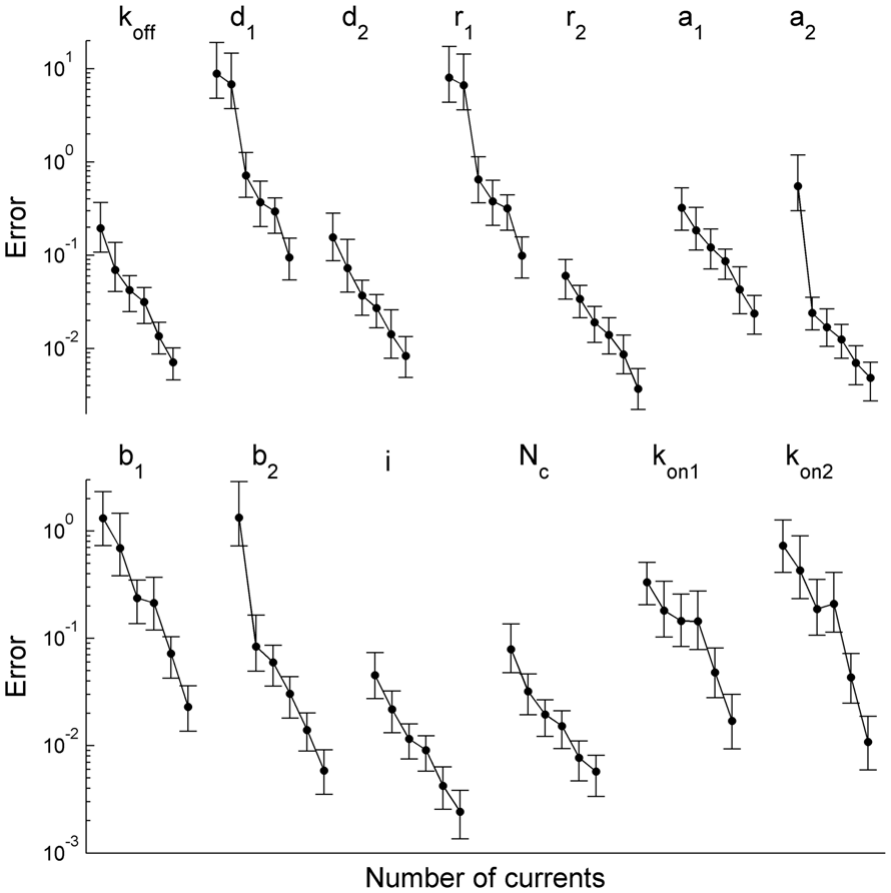
Dependence of relative error of the parameter estimates on the number of currents taken for minimization. Each point represents an averaged (over 26 sets of currents) standard deviation of the estimated parameter from its true value. The number of currents,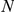, used for the parameter search, increases along each curve from left to right, 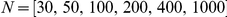. The currents were simulated using the brief stimulation protocol with (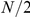 currents) and without (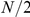 currents) preincubation with GABA. Most parameters, including the channel conductance and the number of channels, were estimated with less than 10% error using only 50 simulated currents.

Parameter estimates (blue lines in Fig. 2) were in good agreement with their true values (green lines in Fig. 2). The relative error of each parameter estimate (i.e., 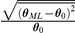, where 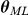 is estimated parameter value), evaluated by bootstrapping (see Methods), was less than 10% (Fig. 3; 4^th^ point of each curve): 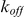 - 1.6%, 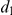 - 10%, 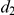
- 3.1%, 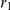 - 12.5%, 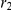 - 0.8%, 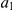 - 8.4%, 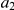 - 0.5%, 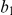 - 6.3%, 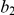 - 1.4%, 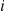 - 0.5%, 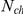 - 0.7%, 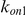 - 4.6% 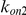 - 4.1%. Relative errors of parameters, estimated using the inverse Hessian matrix of 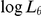 (Eq. 5 in Methods) were in close agreement with their bootstrap estimates: 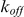 - 1.2%, 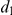 - 17%, 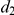
- 1.3%, 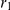 - 17%, 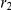 - 1.1%, 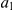 - 3.5%, 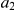 - 1.0%, 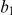 - 6.5%, 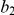 - 0.9%, 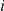 - 0.8%, 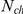 - 1.2%, 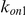 - 2.8%, 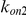 - 2.2%.

**Figure 4:**
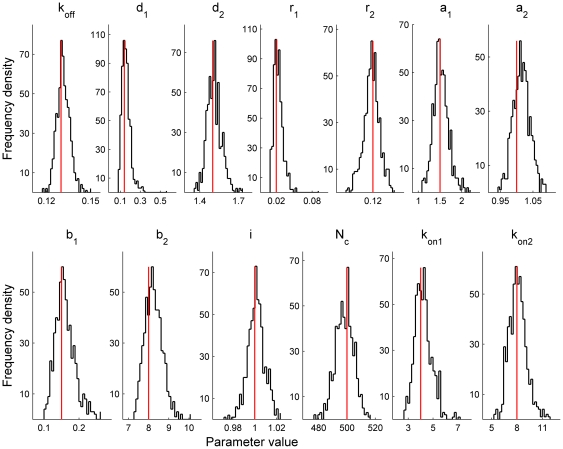
Sampling distributions of model parameter estimates. The graphs show the sampling distribution for each parameter estimate in the case when the combined dataset was used. The parameters are indicated on the top of respective graphs. Red vertical lines mark true parameter values. All distributions are narrow, have a Gaussian shape and centered very close to the true parameter values. Each of 606 parameter searches utilized two sets of simulated currents: 100 currents without and 100 currents with preincubation with 6 µM of GABA, similar to that used in Fig. 1 A–C.

To obtain sampling distributions of estimates, the parameter search was repeated 606 times using the same dataset of 200 currents. Each of the resulting distributions was Gaussian-shaped and narrow, with the sample mean that was very close to the true value (Fig. 4). All estimates, except for 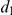, 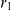, 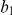, had only slight positive or negative bias of about 1% (calculated as 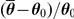). Sampling distributions of these rate constant estimates had coefficients of variation (CV) of about 5%, except for 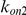 (CV = 13%) and 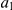 (CV = 12%). Only 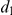, 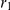, 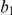 and 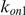 estimates had the sampling distributions with more than 15% CV and the bias of about 4–10%.

### Algorithm Accuracy and Sample Size

The amount of data necessary for a particular algorithm to secure a given accuracy of parameters is an important issue. For example, for synaptic currents it is hard to collect more than a few hundred traces in steady state conditions necessary for applicability of practically any existing algorithm [50]. Therefore, we explored a dependence of accuracy, expressed as a relative error of evaluated parameters on the number of macroscopic currents taken for likelihood maximization (Fig. 3). To this end, 6 groups of currents were selected with each group consisting of 26 sets of randomly sampled currents. The number of currents in these sets, 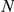, was different, 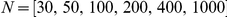, and 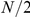 currents were generated using each stimulation protocol as described in the previous section. The model parameters used for generation of the macroscopic currents were estimated for these groups of currents using our maximum likelihood method. Fig. 3 demonstrates that the error of each parameter estimate steadily decreases with the number of currents taken for the analysis. It is also worth noting that parameters 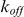, 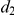, 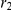, 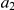, 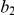, 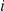, 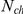 can be evaluated with less than 10% error even from the set of 50 currents. At the same time, parameters 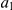, 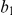, 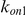, 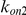 can be estimated from a set of 200–400 currents and only estimation of parameters 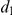, 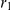 with the same accuracy requires about 1000 currents.

Thus, even in the case of complex channel models our algorithm can accurately evaluate important model parameters such as the channel conductance, the number of channels and kinetic constants determining channel desensitization using experimentally realistic number of macroscopic currents obtained within two simple stimulation protocols.

### Method Accuracy and Sampling Rate

Reduction in the sampling rate, i.e., in the number of data points in each macroscopic current, can significantly speed up the log-likelihood computation, and thus the estimation of the model parameters [22]. An important issue, however, is how the accuracy of the estimates decreases with the reduction of the sampling rate, and how many model parameters could be reliably estimated.

In order to explore this issue, 5 groups of currents having different number of data points were obtained in the following way. Initially 1000 currents with a sampling interval of 0.2 ms were generated using the brief stimulation protocol with (500 currents) and without (500 currents) preincubation with GABA as described in the first chapter of the Results. Thereafter 26 sets of 1000 currents were randomly sampled from these initially generated macroscopic currents. Finally, 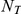 evenly spaced in time data points were selected from these currents with 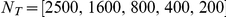 for each group, respectively. The number of data points in the groups of macroscopic currents corresponded to the sampling rates of 
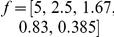
 kHz that are in a range of conventional sampling rates used in electrophysiological recordings of macroscopic currents.

**Figure 5:**
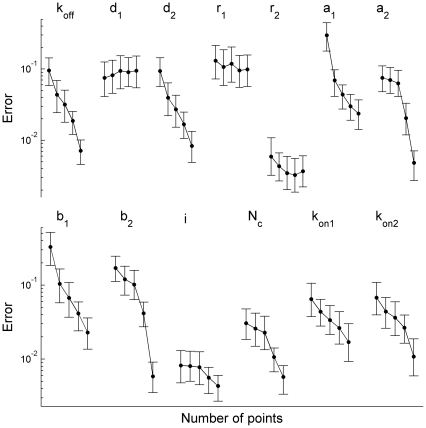
Dependence of relative error of the parameter estimates on the sampling rate. Each point represents an averaged (over 26 sets of 1000 currents) standard deviation of the estimated parameter from its true value. 500 currents without and 500 currents with preincubation with GABA were used. The number of points, *N_t_*, in each of the currents used for the parameter search, increases along each curve from left to right, 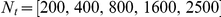, corresponding to the sampling rates of 0.385, 0.83, 1.67, 2.5 and 5.0 kHz, respectively. The accuracy of most parameter estimates steeply depended on the sampling rate.

The parameter search showed that the error of the estimated single-channel conductance, 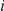, the number of channels, 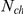, and the rate of escape from the desensitized state, 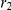, was smaller than 3% and almost independent of the sampling rate. It is also worth noting that most of the other model parameters estimated from the currents sampled at the lowest rate differed from their true values by less than 10%, while some parameters could not be evaluated at all (Fig. 5). At the same time, increasing sampling frequency from 0.385 to 5 kHz significantly improved the accuracy of the estimates of parameters 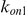, 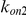, 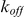, 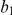 (‘slow’ constants) as well as of 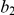, 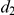, 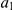, 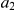 (‘fast’ constants) and 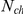 and allowed estimating the parameters that could not be evaluated at lower rates.

Thus, we may conclude that using the method proposed in this work the channel conductance and the number of channels can be efficiently and accurately evaluated from the macroscopic currents recorded at low sampling rates. At the same time the accuracy of almost all parameter estimates significantly improves with the sampling rate increase (regardless of whether the constant is ‘fast’ or ‘slow’), arguing in favor of importance of the method capability to efficiently analyze non-filtered macroscopic currents or to select the large number of data points in the analyzed currents.

### Method Sensitivity to Colored Noise and Open-Channel Excess Noise

In order to test the method behavior under more realistic noise we have conducted computational experiments such that the generated macroscopic currents included colored noise, which was estimated from whole-cell patch clamp recordings, and substantial (10% of single channel current) excess noise (see Macroscopic Current Generator in Methods).

In first series of these experiments the excess noise was modeled as white noise and its standard deviation, 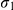, was considered as an additional unknown parameter of the model.

The errors of the parameters evaluated by the method were: 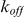 - 5.7%, 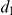 - 30%, 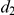
- 7.2%, 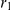 - 36%, 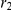 - 1.1%, 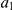 - 11.7%, 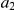 - 10.8%, 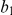 - 17%, 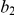 - 8%, 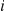 - 2.2%, 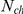 - 4.0%, 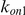 - 12.5% 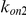 - 11.4%, 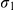-48.2%.

Relative errors of parameters, estimated using the inverse Hessian matrix of 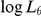 (Eq. 5 in Methods) were: 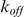 - 5.9%, 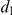 - 26%, 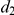
- 7.7%, 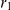 - 26%, 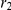 - 1.3%, 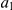 - 11.0%, 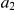 - 11.8%, 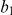 - 13%, 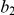 - 10%, 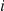 - 2.3%, 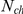 - 4.9%, 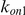 - 10.4% 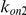 - 9.8%, 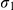-59.2%.

The excess noise, which was modeled in second series of experiments, was thought to arise from fast transitions of the channel between two subconductance states. When this model of excess noise was used the errors of the parameters evaluated by the method were approximately the same as in the first series of experiments: 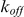 - 5.7%, 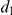 - 21%, 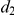
- 8.1%, 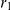 - 25%, 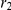 - 1.2%, 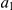 - 11.6%, 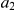 - 11.7%, 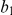 - 17%, 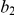 - 8.6%, 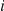 - 1.8%, 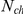 - 3.7%, 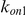 - 11.0% 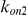 - 15.1%, 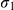 - 50.2%.

Relative errors of parameters, estimated using the inverse of a Hessian matrix of 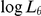 (Eq. 5 in Methods) were: 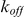 - 6.3%, 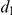 - 21%, 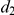
- 8.1%, 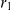 - 21%, 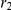 - 1.3%, 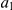 - 10.6%, 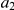 - 11.6%, 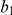 - 14%, 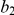 - 10%, 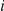 - 1.9%, 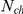 - 4.8%, 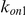 - 9.5% 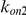 - 11.1%.

Thus, both models were in good agreement with each other, but addition of a realistic noise significantly decreased the accuracy of most parameter estimates. Nevertheless, all parameters can still be estimated with a reasonable accuracy.

All kinetic constants corresponding to double-bound states as well as channel conductance and the number of channels were evaluated with very high accuracy while evaluation of the constants for single-bound states had lower accuracy due to minor representation of O_1_ state in the generated currents. Using a combination of two stimulation protocols, we also succeeded in finding rate constants for transitions R→RG (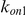) and RG→RG_2_ (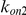) with less than 8% error.

To additionally address a problem of method sensitivity to a signal-to-noise ratio and deviations from Gaussian statistics for the low number of channels [22], the macroscopic currents containing white background noise (3 pA) were generated and analyzed for a set of 50 GABA_A_ channels, the number of channels close to one in a single postsynaptic density. Most parameter estimates were still informative (errors: 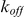 - 9%, 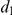 - 105%, 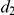
- 9.3%, 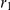 - 72%, 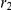 - 1.7%, 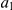 - 24%, 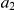 - 7.6%, 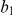 - 73%, 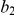 - 19%, 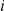 - 1.2%, 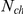 - 2.5%, 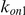 - 58% 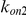 - 42%) indicating that the method could be potentially applicable to the analysis of miniatures and postsynaptic currents recorded from single synapses.

### Evaluation of Fast Kinetic Rates from Noisy Macroscopic Currents

**Figure 6:**
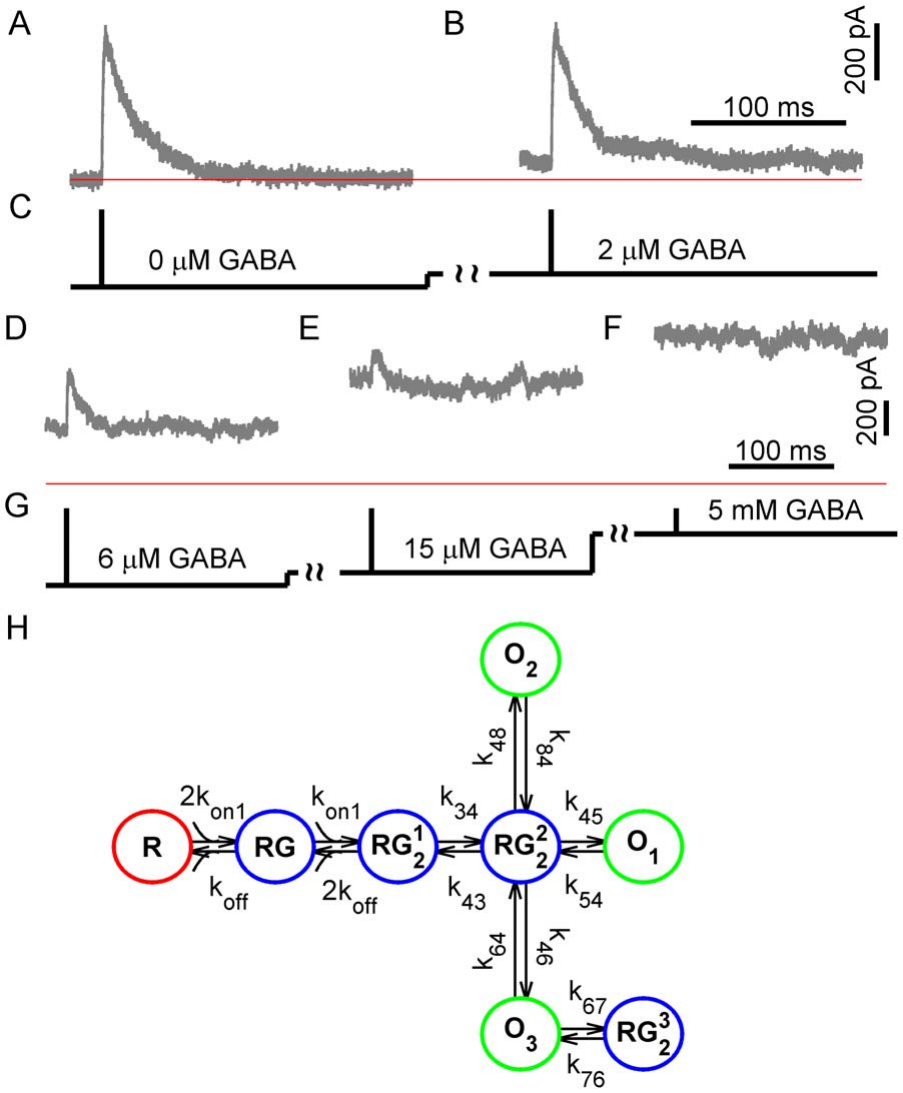
Single-channel analysis based GABA_A_ receptor model, stimulation protocols and simulated currents. Macroscopic currents were simulated by Monte-Carlo method using single-channel analysis based kinetic scheme of GABA_A_ receptor [36]. Complex colored noise with the standard deviation of 3 pA was added to the initially generated currents. (A) Simulated currents produced by a 30-µs application of saturating GABA concentration to 500 unliganded receptors. (B, D–F) Simulated currents evoked by saturating GABA application to receptors that were preincubated with 2, 6, 15 and 5000 µM of GABA, respectively. A thin red horizontal line corresponds to a zero current. (C, G). GABA applications protocols for panels A, B and D–F, respectively. (H) The kinetic model of GABA_A_ receptor used in simulations of macroscopic GABA_A_ receptor current. The model is based on a single-channel data analysis and consists of one single-bound closed state RG, 3 double-bound closed states RG_2_^1^ RG_2_^2^ and RG_2_^3^, and 3 open states O_1_, O_2_ and O_3_. It is suggested that all open states have got the same single-channel current of 3 pA. The published model parameters [41] have been modified by increasing the opening and closing rate constants for one of the channel conducting state (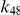 and 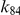) from 1.66 to 66.4 *ms* ^−1^ and from 1.986 to 7.944 *ms* ^−1^, respectively. Other rate constants were as follows: 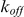 = 0.33, 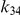 = 0.521, 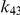 = 1.362, 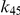 = 1.648, 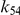 = 0.34, 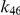 = 0.205, 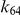 = 0.223, 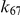 = 1.216, 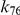 = 0.153, (*ms* ^−1^); 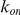 = 17 (*mM* ^−1^•*ms* ^−1^).

The results discussed so far were obtained using the model of GABA_A_ receptor that was originally derived from the analysis of macroscopic currents (Fig. 1 D, [36]). However, other topologies and parameter ranges of GABA_A_ receptor models have been recently obtained based on a single-channel analysis of GABA receptor currents recorded in heterologous systems [41], [51]. For consistency with the previous results, we have chosen to use the recently published GABA_A_ receptor model [41] to test how the presented method performs with a single-channel analysis-based model. The kinetic model topology is shown in Fig. 6. This model was obtained using a segmented K-means method for data idealization that can underestimates fast kinetic rates [52]. Furthermore, the rates of channel gating obtained from single-channel recordings of ligand-gated channels often reach values as high as 130 ms^−1^, as it has been recently shown for nicotinic and glycine receptors [37]–[40]. Considering these facts we modified the model [41] by increasing the opening and closing rate constants for one of the channel conducting states (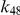 and 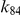) from 1.66 to 66.4 ms^−1^ and from 1.986 to 7.944 ms^−1^, respectively (Fig. 6). Thus, the obtained GABA_A_ receptor model allowed us to test how the presented method performs with models having fast gating kinetics (see Methods).

Realistic colored noise having the root mean square of 12.4 pA was added to these currents. The parameters of noise model were obtained from whole-cell recordings of cultured hippocampal neurons using a sampling interval of 30 µs (see Methods for further details). A total of 250 currents were randomly sampled from 2500 initially generated macroscopic currents for the subsequent analysis.

Relative errors of parameter estimates evaluated by bootstrapping were: 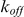 - 3.0%, 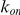 - 1.4%, 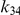
- 3.0%, 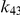 - 4.6%, 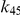 - 24%, 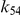 - 16%, 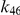 - 9.7%, 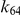 - 15%, 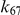 - 9.8%, 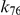 - 7.6% 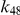 - 3.0%, 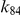 - 9.9%, 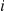 - 2.7%, 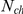 - 4.1%;

Relative errors of parameter estimates evaluated using Hessian inverse were: 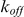 - 2.7%, 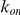 - 1.3%, 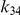
- 2.9%, 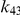 - 4.1%, 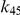 - 24%, 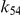 - 15%, 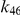 - 8.3%, 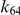 - 13%, 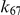 - 9.2%, 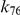 - 7% 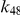 - 3.1%, 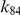 - 8.7%, 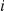 - 2.4%, 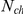 - 3.8%.

**Figure 7:**
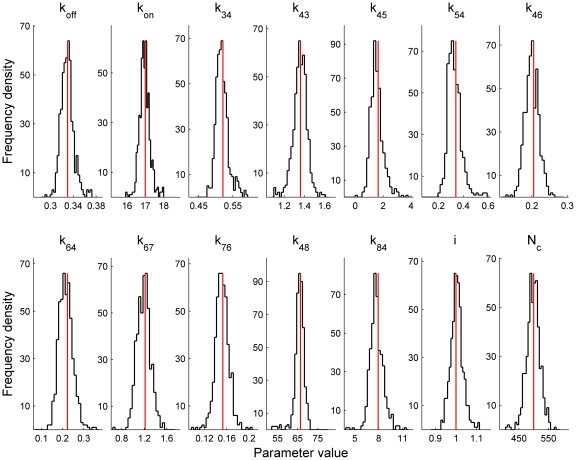
Sampling distributions of parameter estimates for the single-channel analysis-based model. The graphs show the sampling distribution for each parameter estimate. Macroscopic currents were simulated by Monte-Carlo method using a kinetic model of GABA_A_ receptor based on a single-channel data analysis (Fig. 6) and having a fast channel opening rate constant of 66.4 ms^−1^. The simulated currents were produced by a brief (30 µs) application of saturating GABA concentration to 500 GABA_A_ receptors. Complex colored noise was added to the initially generated currents. The estimated model parameters are indicated on the top of each respective graph. Red vertical lines mark true parameter values. Distributions are narrow, Gaussian-shaped and centered very close to the true parameter values. Each of 725 parameter searches utilized 250 simulated currents (see Methods for further details).

Sampling distributions of the estimates were Gaussian-shaped and narrow, with the sample mean that was in each case very close to the true parameter value (Fig. 7). The estimates demonstrated only slight negative bias (calculated as 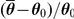) of about 0.5% (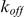, 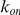, 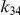, 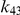, 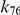, 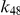, 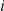, 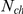) or 3% (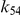, 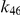, 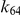, 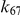 and 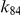) except for 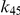, which had 9.5% bias. Sampling distributions of the abovementioned rate constant estimates had coefficients of variation (CV) of about 3% and 12%, respectively. Only 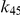 estimate had the sampling distribution with CV = 26%.

Thus, given appropriately recorded and filtered macroscopic currents our method can reliably estimate very fast kinetic rates in realistically complex models, similar to those obtained using the single-channel analysis.

### Algorithm Applicability to Analysis of Synaptic Currents

A protocol with brief channel stimulation by a saturating neurotransmitter concentration that was used for the macroscopic current simulation is a model of synaptic quantal release for a certain type of synapses [34], [43]–[48]. Therefore, we wondered about a possibility to accurately evaluate channel model parameters from macroscopic currents generated with the only brief stimulation protocol instead of two protocols described above.

**Figure 8:**
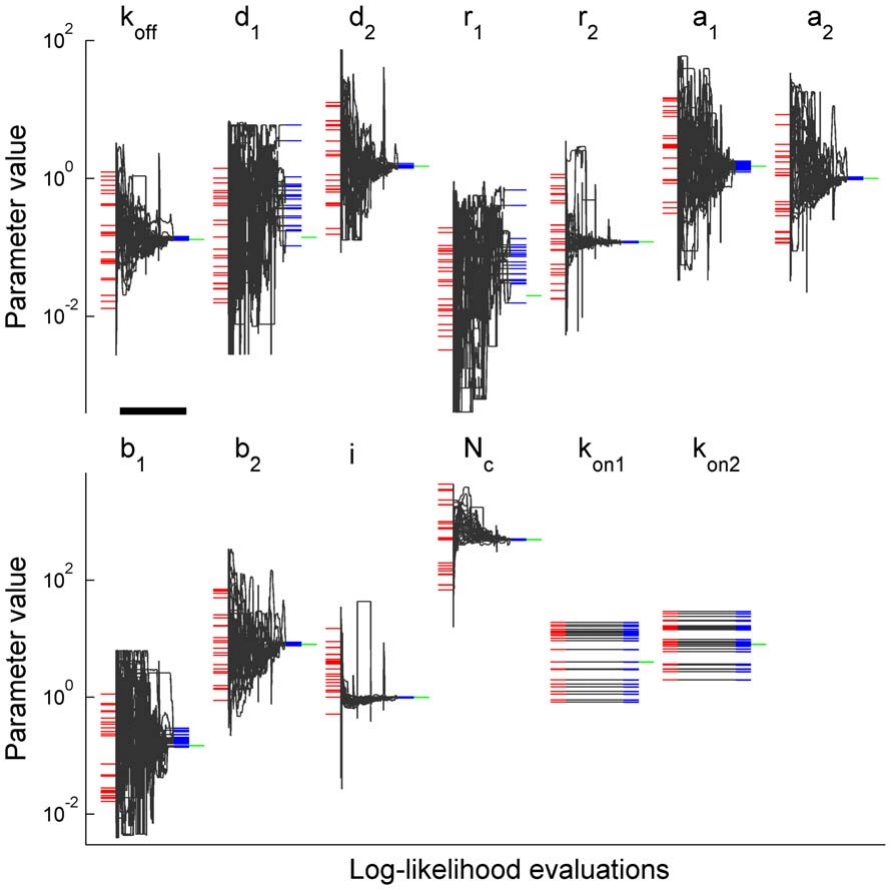
Convergence of the method in the case of brief stimulation. Each of 30 performed parameter searches utilized a set of 200 currents. Each black trace represents the evolution of the respective parameter estimate (indicated on the top) during the maximization of likelihood of a particular set of currents. Each point in the traces represents an iteration of the maximization algorithm. Initial values of each parameter (red dashes) were chosen randomly and uniformly in the logarithmic scale from the interval 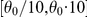, where 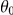 denotes their true values (green dashes). Blue dashes mark parameter values, to which the algorithm converged. The method converged for almost all estimated parameters except of GABA binding constants 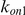, 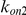 and it showed rather poor convergence for the slow rate constants 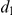 and 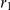. The black horizontal bar corresponds to 2500 log-likelihood evaluations.

Initially, the parameter search was performed using 200 macroscopic currents simulated in a response to the brief pulse of saturating GABA concentration applied to receptors being in the unliganded state. This number of currents is an upper limit of the number of experimental postsynaptic currents that can be routinely recorded in steady state conditions, which are necessary for applicability of MLE methods to the experimental data. The currents were randomly sampled with replacement from the corresponding initially generated set of 1000 currents. In order to determine an accuracy of parameter estimates the parameter search was repeated for 30 sets of macroscopic currents obtained in the manner described above. It appeared that parameters 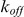, 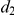, 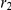, 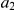, 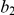, 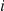 and 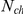 were evaluated with an excellent accuracy (errors: 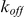 - 2.2%, 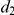
- 2.0%, 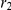 - 1.2%, 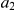 - 1.3%, 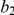 - 2.8%, 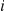 - 0.6%, 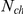 - 1.1%) using 200 macroscopic currents resembling postsynaptic ones (Fig. 8). These particular parameters could also be estimated with a good accuracy already from 50 currents obtained with the brief stimulation protocol (errors: 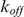 - 4.4%, 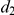
- 5.0%, 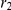 - 2.7%, 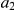 - 3.9%, 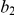 - 7.3%, 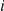 - 1.5%, 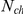 - 2.0%).

Thus, these results open a possibility to substantially reduce the amount of data necessary to secure a given accuracy of the most important channel parameters, that is an important issue in the case of synaptic current studies.

Two constants characterizing transitions between the single-bound open and closed states, 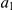 and 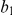, were also identified using 200 macroscopic currents, although less accurately (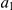 - 6.8%, 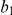 - 21%) than the above-mentioned parameters (Fig. 8). The only parameters that the method failed to identify in the case of brief stimulation protocol were kinetic constants 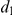, 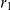, related to the single-bound desensitized state. It is worth noting that kinetic constants 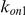 and 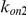 could not be identified using the brief stimulation protocol.

Thus, identification of parameters related to the single-bound states as well as to binding GABA to the receptors requires the second stimulation protocol, whereas other model parameters were identified and accurately estimated in the case of the brief stimulation, implying a possibility to apply the method for analyzing synaptic macroscopic currents.

**Figure 9:**
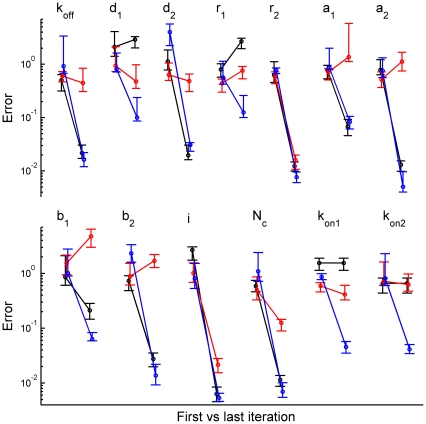
Dependence of relative error of the parameter estimates on the stimulation protocol. Significance of local time correlations. For each parameter (indicated on the top) points on the left correspond to the deviation of its initial values from the true ones; points on the right correspond to the deviation of parameter estimates, evaluated by the respective method, from their true values. Black lines denote that parameters were estimated from a set of 200 currents generated with the brief stimulation protocol without preincubation with GABA. Blue lines denote that parameters were estimated for a set of currents generated using brief stimulation protocol with (100 currents) and without (100 currents) preincubation with GABA. Red lines denote the case when the local time correlations were disregarded (100 currents with and 100 currents without preincubation). In the latter case, the likelihood function was calculated using the covariance matrix with all off-diagonal elements substituted by zeros [21].

Although most of the model parameters were identified by both the brief stimulation protocol and a set of two protocols (the brief stimulation with and without preincubation) it was interesting to compare the algorithm convergence and accuracy of parameter estimates for the experimentally realistic number of macroscopic currents. Results of such comparison for 200 currents are demonstrated in Fig. 9. Points on the left correspond to the deviation of the initial values of each parameter from their true values, and points on the right show the relative error of each parameter estimate. It was found that the algorithm did not converge only for GABA binding constants 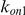, 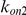 and the error of estimates was significantly larger only for parameters 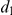, 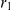, 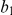 in the case of the brief stimulation protocol compared to the set of two protocols (Fig. 9, black versus blue lines).

As a result we have concluded, that in the case of the simplest stimulation protocol resembling a neurotransmitter concentration profile during a synaptic vesicle release, most kinetic rates of synaptic receptor model, as well as the number of channels and their conductance could be reliably and precisely estimated, and the values for constants related to single-bound states might be restricted to a relatively narrow range. These results raise a possibility that kinetic models of synaptic receptors in their native biochemical environment could be analyzed in detail using routinely recorded macroscopic postsynaptic currents.

### Convergence and Accuracy of MLE Methods Critically Depend on Taking into Account Local Time Correlations

An important advantage of the method, presented in this work, is that it approximates the likelihood of the macroscopic currents including the information contained in local time correlations of the data. We suggested that additional information about channel dynamics, which is present in the local time correlations, might allow for the method to evaluate the model parameters reliably without using complicated stimulation protocols (i.e. the protocol with the large number of concentration or voltage steps).

This could give our method a considerable advantage over ones considering the information about the variance of currents only [21]. This advantage could be especially important for analyzing synaptic receptors when utilizing fast and complicated stimulation protocols are hardly possible. To quantitatively demonstrate this advantage of the method, the convergence and accuracy of parameter estimates were analyzed for our method against the MLE approximation disregarding the local time correlations. To implement the approximated MLE, the calculation of the likelihood function using our method was simplified by substituting zeros for all off-diagonal elements of the covariance matrix of the currents, which is equivalent to the approximation described in [21].

Two groups of currents: (i) 200 currents generated using the brief stimulation protocol and (ii) 200 currents generated using brief stimulation protocols with and without preincubation (100 currents for each protocol) were taken for the analysis. The parameter search was performed for the case of full covariance (our method) for two groups of currents and for the case of the approximated MLE method for the second group of the currents. The search results are demonstrated in Fig. 9 (blue and black lines for our approach versus red lines for the approximated MLE), where points on the left correspond to the deviation of the initial values of each parameter from their true values, and points on the right represent the relative error of each parameter estimate. It is clearly seen that the method presented in this work outperforms the approximated MLE. The approximated MLE provides reliable estimates only for the parameters 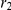, 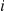 and 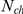, whereas our method could accurately evaluate all model parameters for the same set of two stimulation protocols (blue lines for our approach compared to red lines for the approximated MLE) and identified most of the parameters even in the case of the single stimulation protocol resembling the synaptic vesicle release (black lines for our approach compared to red lines for the approximated MLE). Moreover, the presented method allowed to estimate 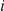 and 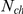 significantly better compared to their estimates obtained with the approximated MLE.

Thus, accounting for the local time correlations provided by our method is crucial for the detailed analysis of ligand-gated channel kinetics using the experimental results obtained with simple stimulation protocols. These protocols (especially the brief stimulation protocol resembling the synaptic vesicle release) are potentially feasible within conventional experimental studies of synaptic receptors and together with our method allow for the analysis and complete identification of receptor model parameters.

## Discussion

In this work we describe a new maximum likelihood method for evaluation of the ion channel rate constants, the number of channels and single channel conductance from macroscopic currents. The macroscopic current is formulated as a non-stationary Gaussian process and the likelihood of the data is maximized with respect to the above mentioned estimated parameters. We have noticed that the covariance matrix of macroscopic currents is quasiseparable. Fast and exact estimation of the likelihood function was performed using this remarkable feature of the covariance. It resulted in developing of the method that takes into account local time correlations and simultaneously scales linearly with the number of channel states, thus efficiently and accurately estimating channel model parameters.

We have also developed a new approach of likelihood gradient evaluation which is almost independent of the number of model parameters and could be used for fast evaluation of kinetic model topology.

The present work was restricted to the analysis of simulated GABA_A_ receptor currents. It was shown that the new method can estimate the channel conductance, the number of channels, and most kinetic constants from the realistic number of simulated GABAergic macroscopic currents using one simple stimulation protocol resembling a synaptic vesicle release, arguing in favor of the method applicability to the analysis of synaptic currents.

### Relative Performance of Different MLE Methods

The main advantage of the current method over the most computationally efficient exact MLE method for Gaussian approximation of macroscopic currents [22], is that it scales linearly with the model complexity, 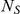, (i.e. with the number of states allowed by the model topology) compared to the cubic dependence on 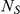 in the MLE method suggested earlier [22].

Indeed, we estimated that the number of operations required by the previous method for the log-likelihood evaluation is approximately equal to 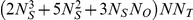 elementary operations (Eq. 46, 47, 50, 51, 54, 55 in [22]), versus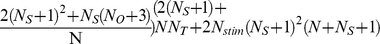 elementary operations (see Eqs. 12–18 in Methods) required by the presented method. It results in a substantial improvement of computational efficiency for complex realistic models. For example, for a standard model of GABA_A_ receptor with 7 states described in this work and macroscopic currents evoked by the simple stimulation protocol, the presented method requires approximately 58 times less elementary operations when a set of 200 macroscopic currents is analyzed. At the same time, both methods calculates likelihood function exactly, resulting in convergence to the same model parameters (for additional comparisons of the presented method with the previous Kalman filter based method [22] see A5 in Text S1).

In addition to the fast calculation of likelihood, we have also developed an approach that allows for fast calculations of log-likelihood gradient. A computational cost of gradient calculation in the suggested approach is weakly dependent on the model complexity and the number of estimated parameters (Efficient Estimation of the Log-likelihood Gradient in Methods and A2 in Text S1). For the sufficiently large number of currents and simple stimulation protocol, the gradient calculation requires approximately 2 times more elementary operations then the calculation of the likelihood function. In contrast, a standard finite difference approach requires 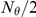 times more operations, where 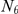 is the number of model parameters (see Eq. 19 for details).

We have also shown that taking into account local time correlations of the macroscopic currents resembling synaptic ones is really important for the convergence and accuracy of parameter estimates in the case of realistic numbers of stimulation protocols and currents. Indeed, the GABA_A_R model parameters have been completely identified and accurately evaluated by our method, using only two simple stimulation protocols. On the other hand, an approximated MLE, that uses the diagonal covariance matrix and ignores the local time correlations [21], can estimate only 3 out of 15 model parameters (Fig. 9). At the same time, the presented method requires 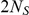 times more operations for the likelihood estimation. However, taking into account that the time required for calculations of parameters of GABA_A_R model described in this work is about 5–10 min (see below), the presented method is not too computationally expensive even compared to the MLE methods ignoring the local time correlations. Thus, we may conclude that the suggested method outperforms ones disregarding local time correlations in terms of required number of macroscopic currents and complexity of stimulation protocols necessary to reach a given accuracy of evaluated parameters.

The covariance fitting approach [20] also fits both the magnitude of the macroscopic current and the strength of the correlation between different time points and uses the full covariance matrix representation as a function of model parameters. However, this approach utilizes log-likelihood approximation by a squared deviation of a model covariance from its statistical estimate, instead of exact calculation,. In addition the method scales as the square of the number of samples and therefore is computationally limited only to subsets of points in the currents. As it was shown above (Fig. 5) the accuracy of parameter estimates constantly and significantly improves up to the sampling rates being as high as 5 kHz when the local time correlations of simulated GABA_A_ receptor currents are taken into account. This strongly argues in favor of analysis of high-frequency current fluctuations that should certainly result in faster and more accurate log-likelihood estimation.

Thus, the presented method evaluates the channel kinetics using both the time course and the random fluctuations of the macroscopic currents, thus securing the maximal accuracy possible with MLE thus far, and simultaneously substantially improves computation efficiency due to faster calculations of the likelihood and likelihood gradient.

### Method Applicability for Arbitrary Stimulation Protocols

The proposed method is applicable to the analysis of both voltage- and ligand-gated ion channels. Its important feature is the ability to perform the parameter search using the macroscopic currents elicited by both simple (no changes in the rate matrix) and complex (arbitrary) stimulation protocols. Continuously changing arbitrary stimuli can be approximated with a series of step functions and the rate matrix for each step is modified by an instantaneous change of those of transition probabilities that depend on a neurotransmitter concentration (if ligand-gated channels are under study) or membrane potential (in a case of voltage-gated channels). At the same time, calculations performed by the method are the most efficient in the case when stimulation protocol consists of a small number of step functions (see Fast Calculation of Log-likelihood in Methods). Additionally we have shown that even the simple protocols might be sufficient for the accurate estimation of ion channel model parameters (Fig. 2, 8, 9).

The performance of the presented approach is good enough to promptly analyze experimental macroscopic currents recorded from a set of ligand- or voltage-gated channels. For example, estimation of 13 parameters of the GABA_A_ receptor model having 7 states from simulated currents (500 channels, 200 currents of 2500 points each) takes about 5–10 min on a 2.8 GHz Intel Core2Duo PC. Additionally, the method can be adapted for the analysis of currents filtered by averaging over a short time interval. This would enable to speed up the estimation of some model parameters without impairing estimates accuracy.

Thus, the method could be an efficient and accurate tool for the analysis of parameters of complex ion channel models, channel interactions with pharmacological agents and modulators, and of other cases when the minimal kinetic scheme includes many states and transitions and when arbitrary stimulation protocols are utilized.

The method can be applied for identifying a broad class of linear dynamical systems beyond the domain of ion channel kinetic model inference. For the complex linear dynamical systems the method can significantly outperform methods based on Kalman filter if i) at least several traces are obtained with each stimulation protocol or ii) a stimulation protocol is relatively simple.

### Kinetic Model Size and Topology Selection

In this study we proceeded from the assumption that the kinetic model topology was known. At the same time, the presented method can give significant advantages in choosing the model size and topology based on differences in the goodness of fit. In perspective, the method could be used in two ways. First, it can substantially increase the quality of evaluation of the model topology when the log-likelihood is used as a score function in the previously described methods [20], [21], [53]–[58].

Second, our method makes it possible to efficiently find kinetic constants even for the complex ion channel models since the log-likelihood gradient computation almost linearly depends on the number of states and is almost independent of the number of transitions between the model states. Thus, it now becomes possible to perform the parameter search, starting from the most complicated model topology, which might be suggested for the particular ion channel and which includes the large number of states and the whole set of theoretically feasible transitions between these states. This search should result in the most likely parameter set, 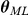, of this complicated model. In the case of the large number of macroscopic currents the likelihood,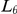, as a function of the parameter vector, 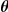, is well approximated by the Gaussian function with the mean equal to 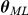 and the inverse of covariance matrix is equal to Hessian of 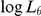, evaluated at the point 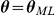. We propose to use this Laplace approximation for the fast search of the most probable model within a set of models having smaller size and simpler topology using Bayesian statistics methods [59].

### Method Applicability to Analysis of Synaptic Currents

The brief channel stimulation with the saturating GABA concentration simulated in this work can be considered as a model of synaptic vesicle release in certain types of synapses. Indeed, after synaptic GABA release from the synaptic vesicle, its concentration in the synaptic cleft decreases by a factor of 10 during less than 0.1 ms [43] and in many types of synapses neurotransmitters almost completely saturate the postsynaptic receptors [34], [44]–[48]. Thus, it is possible that postsynaptic current fluctuations in these synapses occur mainly due to stochastic nature of the respective postsynaptic receptor gating. In this particular case trial-to-trial variations of neurotransmitter concentration in the synaptic cleft [46], [60], and other presynaptic factors [45], [60]–[62] should not contribute to the fluctuations. It is also possible to reach the saturation of postsynaptic receptors in these and other types of synapses by increasing the probability of vesicle release, e.g. using high concentrations of extracellular calcium [34] or by increasing the neurotransmitter content of synaptic vesicles [63]. Thus, in many experimentally conceivable situations a direct application of the presented method to the analysis of macroscopic postsynaptic currents can yield accurate estimates of the channel conductance, the number of channels, and substantial number of kinetic rates (Fig. 8).

However, the information from the macroscopic currents evoked by the synaptic vesicle release could not be enough if a whole set of parameters should be evaluated for a particular ligand-operated synaptic channel (Fig. 9). As it was shown (Fig. 1, 2), all kinetic rates could be accurately estimated only if the currents evoked by synaptic GABA release were combined with an external GABA application to the same synaptic receptors. Although it is technically challenging to apply a neurotransmitter selectively to synaptic receptors, combination of local GABA application (e.g, by iontophoresis or by puff application [64]) with local presynaptic stimulation [65], [66] could potentially resolve this problem. In this case, noise induced by extrasynaptic channel activation can be estimated from currents induced by the local neurotransmitter application after blocking of synaptic currents with an irreversible use-dependent inhibitor of the synaptic receptors (e.g, picrotoxin for GABA_A_ or MK-801 for NMDA receptors, respectively [67], [68]). Other approach could be a combination of temporary use-dependent block of synaptic channels with competitive irreversible block of extrasynaptic receptors. The GABA concentration within the synaptic cleft during applications can be calibrated based on the dependence of postsynaptic current amplitude on the known bath applied GABA concentration.

In this study we have shown that the newly introduced method can accurately evaluate parameters of synaptic receptor model under conditions of saturation of the fixed number of postsynaptic receptors. At the same time, it seems possible to modify the described method in order to apply it for the analysis of any postsynaptic current, i.e. for the case when each postsynaptic receptor is potentially subjected to a different brief neurotransmitter profile in each particular trial. Then, the number of channels in single and double bound states, 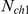 and 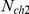, could be estimated separately for each current, 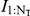, by means of minimization of each 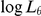 with respect to these additional parameters. Then, 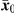 in Eqs. 17 should be modified to utilize 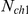, 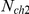 instead of 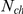. Thus, such an improvement of the method might lead to the development of the algorithm suited for accurate model analysis of any types of synaptic receptors using routinely recorded macroscopic postsynaptic currents.

## Supporting Information

Text S1 **Appendix.** A0 Mean and Covariance as a Function of Kinetic Model Parameters. A1 Linear Algebra Algorithms for Semiseparable Matrices. A2 Log-likelihood Gradient. A3 Log-likelihood Calculation in the Case of Complex Protocol. A4 Method Implementation. A5 Comparison of Different Algorithms for Log-likelihood Evaluation.
(DOC)
