# Supplementary material for: Efficient Maximum Likelihood Estimation of Kinetic Rate Constants from Macroscopic Currents
Source: PLoS One. 2011 Dec 29;6(12):e29731. doi: 10.1371/journal.pone.0029731 (PMC3248447; doi:10.1371/journal.pone.0029731)
Supplement: Text S1 — Appendix. A0 Mean and Covariance as a Function of Kinetic Model Parameters. A1 Linear Algebra Algorithms for Semiseparable Matrices. A2 Log-likelihood Gradient. A3 Log-likelihood Calculation in the Case of Complex Protocol. A4 Method Implementation. A5 Comparison of Different Algorithms for Log-likelihood Evaluation. (DOC) [file pone.0029731.s001.doc]

## Appendix

All linear algebra algorithms described here were taken from or derived on the basis of the work of Eidelman and Gohberg [27].

Throughout the text we use the following notation: for matrices we use bold font, e.g. $\mathbf{q}$; The i-th row or column of the matrices is denoted by the same letter but font is not bold and italic, *e.g.* $q_{i}$; matrices that are the sum of $\text{i}$ matrices - bold and with the upper index $\text{i}$, corresponding to summation, e.g. $\mathbf{f}^{\mathbf{i}}$. Operation $\nabla\mathbf{a}$, where $\mathbf{a}$ is a matrix, denotes a tensor of rank 3 with elements given by $\left\{\nabla\mathbf{a}\right\}_{ijs}=\frac{\partial\left\{\mathbf{a}\right\}_{ij}}{\partial\theta_{s}}$.

## A0

## Mean and Covariance as a Function of Kinetic Model Parameters

Let’s introduce a state probability vector$\text{p}(\text{t})$ to describe the occupancy of each of $N_{S}$ conformational states of a channel at time point $t$. Then $\text{p}_{i}(\text{t})$ is the probability to find a channel in the state $i$ at time point $t$. Now we can write down mean, $E\left(\text{x}_{\text{t}}\right)$, and covariance, $\mathbf{c}_{m}$, of the vector $x$ (see Asymptotic Log-likelihood chapter of Methods for definition) in terms of the state probability vector. To write an expression for the covariance, we note that the product $x_{t}x_{t'}$ differs from zero only in the case when the channel was open at both time points $t$ and $t'$. Thus, we have

$\text{E}\left(\text{x}_{\text{t}}\right)=\sum_{\text{o}=1}^{\text{N}_{\text{O}}} \text{p}_{\text{o}}\left(\text{t}\right)$ (A0.1)

$\left\{\mathbf{c}_{\text{m}}\right\}_{t,t'}=\text{E}\left(\text{x}_{\text{t}}\text{x}_{\text{t'}}\right)-\text{E}\left(\text{x}_{\text{t}}\right)\text{E}\left(\text{x}_{\text{t'}}\right)=\sum_{\text{o}=1}^{\text{N}_{\text{O}}} \text{p}_{\text{o}}\left(\text{t}\right)\sum_{\text{o'}=1}^{\text{N}_{\text{O}}} \text{p}_{\text{o',o}}(\text{t'|t})-\sum_{\text{o}=1}^{\text{N}_{\text{o}}} \text{p}_{\text{o}}\left(\text{t}\right)\sum_{\text{o}=1}^{\text{N}_{\text{o}}} \text{p}_{\text{o}}\left(\text{t'}\right)$ (A0.2)

where $p_{o}\left(t\right)$ is the probability that the channel is in the $o$-th open state at time point $t$,

$\text{p}_{\text{o',o}}(\text{t'|t})$ is the conditional probability that the channel is in the open state $o'$ at time $t'$ given it was in the open state $o$ at time $t$; $N_{O}$ is the total number of open states in the model. Thus, for the elements of mean and covariance of the single-channel current we have:

$\begin{matrix} \text{μ}_{\text{t}}^{1}=\text{i}_{\text{t}}\text{x}_{\text{t}}=\sum_{\text{o}=1}^{\text{o}=\text{N}_{\text{o}}} \text{i}_{\text{o}}\text{p}_{\text{o}}\left(\text{t}\right) \\ \left\{\mathbf{c}_{\text{m}}^{1}\right\}_{\text{t,t'}}=\text{E}\left(\text{i}_{\text{t}}\text{x}_{\text{t}}\text{i}_{\text{t'}}\text{x}_{\text{t'}}\right)-\text{μ}_{\text{t}}\text{μ}_{\text{t'}}=\sum_{\text{o}=1}^{\text{N}_{\text{O}}} \text{i}_{\text{o}}\text{p}_{\text{o}}\left(\text{t}\right)\sum_{\text{o'}=1}^{\text{N}_{\text{O}}} \text{i}_{\text{o'}}\text{p}_{\text{o',o}}\left(\text{t'|t}\right)-\text{μ}_{\text{t}}\text{μ}_{\text{t'}} \end{matrix}$ (A0.3)

And for the elements of mean and covariance of the sum of single-channel currents, mediated by $N_{ch}$ identical and statistically independent channels, we have:

$\begin{matrix} \text{μ}_{\text{t}}=\text{N}_{\text{ch}}\sum_{\text{o}=1}^{\text{N}_{\text{O}}} \text{i}_{\text{o}}\text{p}_{\text{o}}\left(\text{t}\right) \\ \left\{\mathbf{c}_{\text{m}}\right\}_{\text{t,t'}}=\text{N}_{\text{ch}}\sum_{\text{o}=1}^{\text{N}_{\text{O}}} \text{i}_{\text{o}}\text{p}_{\text{o}}\left(\text{t}\right)\sum_{\text{o'}=1}^{\text{N}_{\text{O}}} \text{i}_{\text{o'}}\text{p}_{\text{o',o}}\left(\text{t'|t}\right)-\frac{\text{μ}_{\text{t}}\text{μ}_{\text{t'}}}{\text{N}_{\text{ch}}} \end{matrix}$ (A0.4)

To estimate likelihood (Eq. 1 in Methods) as a function of model parameters, we need to express $\text{p}_{\text{o}}\left(\text{t}\right)\text{,}\text{p}_{\text{o',o}}\left(\text{t'|t}\right)$ in terms of the rate constants $k_{ij}$. To this end we introduce a rate matrix [18, 20], $\mathbf{Q}=\{\text{k}_{\text{ij}}\}$, which is composed of rate constants according to the following rules: each off-diagonal element, $k_{ij}=\lim_{\Delta t\to0} \frac{p_{ij}}{\Delta t}$, is equal to the rate constant of the transition from the state $j$ to the state $i$, if the transition is allowed by the model. Otherwise, $k_{ij}=0$. The diagonal elements, $\text{k}_{ii}$, are set to $-\sum_{j} \text{k}_{\text{ij}}$, so the sum over each column is zero. For convenience, the matrix $\mathbf{Q}$ is ordered so that its first $N_{O}$ columns and rows correspond to conducting states of the channel.

The evolution of the state probability vector$p$ satisfies Kolmogorov equation , integrating which we can write down the probability $p_{o}(t)$ and the conditional probability $\text{p}_{\text{o',o}}(\text{t'|t})$ as

$\begin{matrix} p_{o}(t)=\left\{e^{\mathbf{Q}t}\right\}_{o,:}p(0) \\ p_{o',o}(t'|t)=\left\{e^{\mathbf{Q}(t'-t)}\right\}_{o,o'} \end{matrix}$ (A0.5)

where $p_{j}\left(0\right)$ is the probability that the channel initial state is$j$. This initial state vector can be expressed as a function of kinetic model using Eq. A0.5 provided the concentration/voltage stimulation applied to the channels is known during sufficiently long time preceding current registration (i.e. $p(0)=\prod_{j} e^{\mathbf{Q}_{j}\Delta t_{j}}p(-T)$).

Substitution of Eqs. A0.5 into the Eqs. A0.4 gives the well-known equations for the mean and covariance of macroscopic current as a function of a kinetic model [18] (Eqs. 2 in Methods).

$\begin{matrix} \mu_{t}=N_{ch}i^{T}e^{\mathbf{Q}t}p(0) \\ \left\{c_{m}\right\}_{t,t'}=N_{ch}\left(i^{T}e^{\mathbf{Q}t}p(0)e^{\mathbf{Q}\left(t'-t\right)}i-\left(i^{T}e^{\mathbf{Q}t'}p(0)\right)\left(i^{T}e^{\mathbf{Q}t}p(0)\right)\right) \end{matrix}$ (A0.6)

In the case of complex stimulation protocol let a kinetic model be described by the matrix $\mathbf{Q}_{\mathbf{s}}$ during a stimulation protocol step $\text{s}$ that lasted from time $T_{s-1}$ to time $T_{s}$. Then the solution to the Kolmogorov equation at a time $\text{t}$ is:

$\text{p}\left(\text{t}\right)=\text{e}^{\mathbf{Q}_{\mathbf{s}}(t-T_{s-1})}\left(\prod_{\nu=1}^{s-1} \text{e}^{\mathbf{Q}_{\mathbf{\nu}}(T_{\nu}-T_{\nu-1})}\right)\text{p}\left(0\right)$ (A0.7)

where $\text{e}^{\mathbf{Q}_{\mathbf{\nu}}t}=\mathbf{U}_{\nu}\text{e}^{\mathbf{D}_{\nu}t}\mathbf{U}_{\nu}^{-1}$ and $\mathbf{D}_{\nu}$ is a diagonal form of $\mathbf{Q}_{\nu}$ provided all eigenvalues $\lambda_{\nu}$ are different.

It follows from this that:

$\text{p}_{\text{o}}\left(\text{t}\right)=\sum_{j=1}^{N_{S}} \text{U}_{\text{s}}\left(\text{o,j}\right)\text{e}^{\lambda_{s,j}(t-T_{s-1})}\left\{\mathbf{U}_{s}^{-1}\left(\prod_{\nu=1}^{s-1} \text{e}^{\mathbf{Q}_{\mathbf{\nu}}(T_{\nu}-T_{\nu-1})}\right)\text{p}\left(0\right)\right\}_{j}$ (A0.8)

$\text{p}_{\text{o',o}}\left(\text{t|t'}\right)=\sum_{j=1}^{N_{S}} \text{U}_{\text{s}}\left(\text{o,j}\right)\text{e}^{\lambda_{s,j}(t-T_{s-1})}\mathbf{U}_{s}^{-1}\left(\prod_{\nu=s'+1}^{s-1} \text{e}^{\mathbf{Q}_{\mathbf{\nu}}(T_{\nu}-T_{\nu-1})}\right)\mathbf{U}_{s'}\text{e}^{\lambda_{s,j}(T_{s'}-t')}\text{U}_{\text{s'}}^{-1}\left(\text{j,o'}\right)$(A0.9)

where $p_{o',o}(t|t')$ is the conditional probability that the channel is in the open state $o$ at time $t$ given it was in the open state $\text{o'}$ at time $\text{t'}$.

It follows from Eqs. A0.4, A0.8, A0.9 that in the case of a complex protocol $\mathbf{c}_{\mathbf{m}}$ is given by:

$\begin{matrix} \left\{\text{c}_{\text{m}}\right\}_{ij}=\sum_{k=1}^{N_{S}+1} \text{A}_{ik}\left(\prod_{\nu=s\left(j\right)+1}^{s(i)-1} \text{e}^{\mathbf{Q'}_{\mathbf{\nu}}(T_{\nu}-T_{\nu-1})}\right)\text{B}_{kj},\text{i}\geq\text{j} \\ \left\{\text{c}_{\text{m}}\right\}_{ji}=\left\{\text{c}_{\text{m}}\right\}_{ij},\text{i}\geq\text{j} \end{matrix}$ (A0.10)

where matrices $\mathbf{A}$,$\mathbf{B}$ and $\text{e}^{\mathbf{Q'}_{\mathbf{\nu}}t}$ satisfy the following equations

$\begin{matrix} \text{A}_{\text{ik}}=\text{e}^{\text{λ}_{\text{s(i),k}}\text{t}_{\text{i}}}\sum_{o=1}^{\text{N}_{\text{O}}} \text{i}_{\text{o}}\text{U}_{\text{s}\left(\text{i}\right)}\left(\text{o,k}\right)\text{,}1\leq\text{k}\leq\text{N}_{\text{S}} \\ \text{A}_{\text{ik}}=\frac{\mu_{\text{t}_{\text{i}}}}{\text{N}_{\text{ch}}}\text{,}\text{k}=\text{N}_{\text{S}}+1 \end{matrix}$ (A0.11)

$\begin{matrix} \text{B}_{\text{kj}}=\text{N}_{\text{ch}}\text{e}^{\text{λ}_{\text{s(j),k}}\left(\text{T}_{\text{s(j)}}-\text{t}_{\text{j}}\right)}\left(\sum_{o'=1}^{\text{N}_{\text{O}}} \text{U}_{\text{s}\left(\text{j}\right)}^{-1}\left(\text{k,o'}\right)\text{p}_{\text{o'}}\left(\text{t}_{\text{j}}\right)\text{i}_{\text{o'}}\right)\text{,}1\leq\text{k}\leq\text{N}_{\text{S}} \\ \text{B}_{\text{kj}}=-\text{μ}_{\text{t}_{\text{j}}}\text{,}\text{k}=\text{N}_{\text{S}}+1 \end{matrix}$ (A0.12)

$e^{\mathbf{Q}'_{\mathbf{\nu}}t}=\left|\begin{matrix} e^{\mathbf{Q}_{\mathbf{\nu}}t} & 0 \\ 0 & 1 \end{matrix}\right|$ (A0.13)

Thus, is quasiseparable matrix.

## А1

## Linear Algebra Algorithms for Semiseparable Matrices

Consider a symmetric semiseparable matrix $\mathbf{c}_{\mathbf{m}}$ which have constituent matrices $\mathbf{p}=\left|-p_{j}-\right|$ and $\mathbf{q}=\left|\begin{matrix} | \\ q_{i} \\ | \end{matrix}\right|$ and diagonal matrix $d_{ii}=d_{i}$ of dimensions $N_{t}\times(N_{S}+1)$, $(N_{S}+1)\times N_{t}$, $N_{t}\times N_{t}$, respectively.

Cholesky Decomposition

Assume that $\mathbf{c}_{\mathbf{m}}$ can be represented as :

$\mathbf{c}_{\mathbf{m}}=\mathbf{GDG}^{T}$, (А1.1)

where $\mathbf{G}$ is lower triangular, $\mathbf{G}_{ij}=0$, $i<j$, $\mathbf{G}_{ii}=1$ and $\mathbf{D}$ is diagonal matrix, $\mathbf{D}_{ii}=g_{i}$. Then $\mathbf{G}$ is also semiseparable with components $\mathbf{G}_{ij}=p1_{i}q1_{j}$

$\begin{matrix} \left\{\mathbf{c}_{\mathbf{m}}\right\}_{ij}=\sum_{k=1}^{j} \mathbf{G}_{ik}\mathbf{G}_{jk}g_{k}=\sum_{k=1}^{j} p1_{i}q1_{k}p1_{j}q1_{k}g_{k}=p1_{i}\left(\sum_{k=1}^{j} q1_{k}q1_{k}^{T}g_{k}\right)p1_{j}^{T},i,j=1:N_{t},i>j \\ d_{j}\equiv\left\{\mathbf{c}_{\mathbf{m}}\right\}_{jj}=p1_{j}\left(\sum_{k=1}^{j} q1_{k}q1_{k}^{T}g_{k}\right)p1_{j}^{T},\begin{matrix} & j>1 \end{matrix} \\ g_{1}=d_{1} \end{matrix}$(А1.2)

Since $\left\{\mathbf{c}_{\mathbf{m}}\right\}_{ij}=p_{i}q_{j}$ it is clear that the matrix $\mathbf{G}$ is indeed semiseparable with components $p1_{i}$ and $q1_{i}$ given by equations:

$\begin{matrix} p1_{i}=p_{i} \\ \mathbf{f}^{\mathbf{j}}p1_{j}^{T}=q_{j} \\ \mathbf{f}^{\mathbf{j}}=\left(\sum_{k=1}^{j} q1_{k}q1_{k}^{T}g_{k}\right) \end{matrix}$ (А1.3)

This yields that the matrix $\mathbf{f}^{j}$ can be calculated recursively

(A1.4)

Taking into account that $\mathbf{G}_{jj}=1$, recurrent relations for $q1$ and $g$ then follow from Eq. А1.3 and А1.4,

$\begin{matrix} q1_{j}=\frac{q_{j}-\mathbf{f}^{j-1}p_{j}^{T}}{g_{j}},\begin{matrix} & N>j>1 \end{matrix} \\ g_{j}=d_{j}-p1_{j}\mathbf{f}^{j-1}p1_{j}^{T},\begin{matrix} & N\geq j>1 \end{matrix} \\ g_{1}=d_{1} \end{matrix}$ (A1.5)

Equations А1.4, А1.5 give an algorithm for computing Cholesky factorization of a semiseparable matrix.

Solution of a System of Linear Equations

Calculation of log-likelihood also includes procedure of solving the system of equations which has the form (Eq. 11 in Methods)

$\mathbf{y}=\mathbf{Gx}$ (A1.6)

where $\mathbf{G}$ is lower triangular and semiseparable matrix of coefficients, $\mathbf{x}$ and $\mathbf{y}$ are $N_{t}\times N$ matrices. To solve this system, we obtain recurrent relations similar to Eqs. А1.4, А1.5. Indeed,

$\begin{matrix} y_{i}=\sum_{j=1}^{i} \mathbf{G}_{ij}x_{j}=\left(\sum_{j=1}^{i-1} p1_{i}q1_{j}x_{j}\right)+\mathbf{G}_{ii}x_{i}=p1_{i}\mathbf{z}^{i-1}+x_{i} \\ \mathbf{z}^{i}=\sum_{j=1}^{i} q1_{j}x_{j}=\mathbf{z}^{i-1}+q1_{i}x_{i} \end{matrix}$ (А1.7)

where $x_{j},y_{i}$ stand for the row-vector of the length $N$ and $i,j=1:N_{t}$. Eq. А1.7 yields

$\begin{matrix} x_{i}=y_{i}-p1_{i}\mathbf{z}^{\mathbf{i}-\mathbf{1}},\begin{matrix} & i>1 \end{matrix} \\ x_{1}=y_{1} \end{matrix}$ (A1.8)

For the system of equations with the upper triangular and semiseparable matrix of coefficients

$y=\mathbf{G}^{T}x$ (A1.9)

the relations similar to Eq. A1.7 are

and they yield recurrent relations

(А1.10)

Computing log-likelihood of the set of ion-channel currents, we have to deal with semiseparable matrices whose constituent vectors have the form

$\begin{matrix} p_{i}=p'_{i}\circ e^{\lambda^{T}t_{i}} \\ q_{i}=q'_{i}\circ e^{-\lambda t_{i}} \end{matrix}$ (A 1.11)

Here denotes Hadamard multiplication, i.e. elementwise matrix multiplication, $\lambda$ is a column-vector composed of eigenvalues of $Q$ (see A0). For large $t_{i}$ it is impossible to represent such elements as a floating point numbers. To address this problem we have modified algorithms А1.4, А1.7, А1.10, so that matrices $\mathbf{p'}$ and $\mathbf{q}'$appear in them instead of matrices $\mathbf{p}$ and $\mathbf{q}$. Indeed, it is easy to check that the algorithm А1.7, А1.8 can be represented as

$\begin{matrix} \mathbf{z1}^{\mathbf{i}}=\left(\mathbf{z1}^{\mathbf{i}-\mathbf{1}}+q1'_{i}x_{i}\right)\circ\left(\exp\left(\lambda\Delta t\right)\cdot1_{N}^{T}\right) \\ x_{i}=y_{i}-p1'_{i}\mathbf{z1}^{\mathbf{i}-\mathbf{1}},i>1 \\ x_{1}=y_{1} \end{matrix}$ (А1.12)

where $\mathbf{z1}^{\mathbf{i}}=\mathbf{z}^{\mathbf{i}}\circ\left(\exp\left(\lambda t_{i+1}\right)\cdot1_{N}^{T}\right)$, $1_{N}$ is a vector of the size $N$, whose elements are ones, so that $\exp\left(\lambda\Delta t\right)\cdot1_{N}^{T}$ is $N_{S}\times N$ matrix and $t_{i}=i\Delta t$.

The algorithm А1.10 is represented as

$\begin{matrix} x_{N}=y_{N} \\ \mathbf{z1}^{\mathbf{i}}=\left(\mathbf{z1}^{\mathbf{i}+\mathbf{1}}+q1'_{i+1}x_{i+1}\right)\circ\left(\exp\left(-\lambda\Delta t\right)\cdot1_{N}^{T}\right),i<N \\ x_{i}=y_{i}-p1'_{i}\mathbf{z1}^{\mathbf{i}},i>1 \end{matrix}$ (A1.13)

The algorithm expressed in Eqs. А1.4, А1.5 is represented as

(A1.14)

where $\mathbf{f1}^{j}=\mathbf{f}^{j}\circ\left(\exp\left(\left(j+1\right)\lambda\Delta t\right)\cdot\exp\left(\left(j+1\right)\lambda\Delta t\right)^{T}\right)$

Next, we consider few additional operations with semiseparable matrices, which allow for efficient computation of the log-likelihood gradient.

Symmetric Semiseparable Matrix Inversion

First, consider the problem of finding $\mathbf{G}$ inverse. This matrix is a solution of the system $\mathbf{GG}^{-1}\equiv\mathbf{Gx}=\mathbf{E}$ where $\mathbf{E}$ is identity matrix. Because $\mathbf{G}$ is lower triangular and $\mathbf{G}_{jj}\equiv d_{j}=1$, we try the solution of the above equation in the form of a lower triangular matrix: $\mathbf{x}_{ij}=0$ for $i<j$, $\mathbf{x}_{jj}=1$. Thus,

$\sum_{k=j}^{i-1} p1_{i}q1_{k}\mathbf{x}_{kj}+d_{i}\mathbf{x}_{ij}=\mathbf{y}_{ij}=0$ for $i>j$ (A1.15)

Therefore,

$\mathbf{x}_{ij}=-\sum_{k=j}^{i-1} p1_{i}q1_{k}\mathbf{x}_{kj}$ (A1.16)

For $\mathbf{x}_{i+1,j}$ we have

$\mathbf{x}_{i+1,j}=-p1_{i+1}\sum_{k=j}^{i-1} q1_{k}\mathbf{x}_{kj}-p1_{i+1}q1_{i}\mathbf{x}_{ij}=-p1_{i+1}\left(\mathbf{E}-q1_{i}p1_{i}\right)\sum_{k=j}^{i-1} q1_{k}\mathbf{x}_{kj}$ (A1.17)

Continuing transformation, we obtain

$\mathbf{x}_{i+1,j}=-p1_{i+1}\left(\mathbf{E}-q1_{i}p1_{i}\right)\left(\mathbf{E}-q1_{i-1}p1_{i-1}\right)\cdots\left(\mathbf{E}-q1_{j+1}p1_{j+1}\right)q1_{j}$ (A1.18)

Therefore, $\mathbf{G}^{-1}$ is a lower triangular matrix and it can be represented as

$\begin{matrix} \{\mathbf{G}^{-1}\}_{ij}=p2_{i}\mathbf{a}_{i-1}\mathbf{a}_{i-2}...\mathbf{a}_{j+1}q2_{j} \\ \{\mathbf{G}^{-1}\}_{ii}=d2_{i}=1 \end{matrix}$, (А1.19)

where $p2=-p1$, $\mathbf{a}_{i}=\mathbf{E}-q1_{i}p1_{i}$, $q2=q1$

The matrix whose off-diagonal elements are given by Eq. А1.19 (with arbitrary matrices $\mathbf{a}_{i}$and diagonal elements) is called quasiseparable. Thus, $\mathbf{G}^{-1}$ is quasiseparable.

Now we find the matrix $\mathbf{c}_{\mathbf{m}}^{-\mathbf{1}}$

$$\{\mathbf{c}_{\mathbf{m}}^{-\mathbf{1}}\}_{ij}=\sum_{k=i+1}^{N_{T}} \{\mathbf{G}^{-1}\}_{ki}\{\mathbf{G}^{-1}\}_{kj}g_{k}^{-1}+\{\mathbf{G}^{-1}\}_{ij}\{\mathbf{G}^{-1}\}_{ii}g_{i}^{-1}=\left(\sum_{k=i+1}^{N_{T}} g_{k}^{-1}p2_{k}\mathbf{a2}_{ki}q2_{i}p2_{k}\mathbf{a2}_{k,i-1}\right)\mathbf{a}2_{ij}q2_{j}+p2_{i}\mathbf{a2}_{ij}q2_{j}g_{i}^{-1}$$

where $\mathbf{a2}_{ij}=\mathbf{a}_{i-1}\mathbf{a}_{i-2}...\mathbf{a}_{j+1}$ and the following relations hold: $\mathbf{a2}_{ij}=\mathbf{a}_{j+1}\mathbf{a2}_{i(j+1)}$, $\mathbf{a2}_{(i+1)i}=\mathbf{E}$

The first term on the right side can be rearranged so that the equation appear as

$\{\mathbf{c}_{\mathbf{m}}^{-\mathbf{1}}\}_{ij}=p3_{i}\mathbf{a2}_{ij}q2_{j}+p2_{i}\mathbf{a2}_{ij}q2_{j}g_{i}^{-1}=p5_{i}\mathbf{a2}_{ij}q2_{j}$ (А1.20)

where

$\begin{matrix} p3_{i}=\sum_{k=i+1}^{N_{T}} p4_{ki}q2_{i}p4_{ki}g_{k}^{-1}\mathbf{a}_{i}=q2_{i}^{T}\left(\sum_{k=i+1}^{N_{T}} g_{k}^{-1}p4_{ki}^{T}p4_{ki}\right)\mathbf{a}_{i}, \\ p4_{ki}=p2_{k}\mathbf{a2}_{ki},\begin{matrix} & p5_{i} \end{matrix}=p3_{i}+p2_{i}g_{i}^{-1} \end{matrix}$ (А1.21)

We introduce auxiliary variable matrix

$\mathbf{f}_{i+1}=\sum_{k=i+1}^{N_{T}} g_{k}^{-1}p4_{ki}^{T}p4_{ki}$ (А1.22)

so that

$p3_{i}=q2_{i}^{T}\mathbf{f}_{i+1}\mathbf{a}_{i}$ (А1.23)

It satisfies the following recurrent relations

$$\begin{matrix} \mathbf{f}_{i}=g_{i}^{-1}p2_{i}^{T}p2_{i}+\sum_{k=i+1}^{N_{T}} g_{k}^{-1}\left((p2_{k}\mathbf{a2}_{ki}\mathbf{a}_{i})^{T}p2_{k}\mathbf{a2}_{ki}a_{i}\right)=g_{i}^{-1}p2_{i}^{T}p2_{i}+\sum_{k=i+1}^{N_{T}} g_{k}^{-1}\left(\mathbf{a}_{i}^{T}\mathbf{a2}_{ki}^{T}p2_{k}^{T}p2_{k}\mathbf{a2}_{ki}\mathbf{a}_{i}\right)= \\ =g_{i}^{-1}p2_{i}^{T}p2_{i}+\mathbf{a}_{i}^{T}\mathbf{f}_{i+1}\mathbf{a}_{i} \\ \\ \mathbf{f}_{N_{T}}=g_{N_{T}}^{-1}p2_{N_{T}}^{T}p2_{N_{T}} \end{matrix}$$

After substitution of $\mathbf{a}_{i}$, we have the recurrent equation for $\mathbf{f}$:

$\mathbf{f}_{i}=g_{i}^{-1}p2_{i}^{T}p2_{i}+\mathbf{f}_{i+1}-pq\mathbf{f}-\left(pq\mathbf{f}\right)^{T}+pq\mathbf{f}qp$ (А1.24)

where

$$\begin{matrix} pq\mathbf{f}=p1_{i}^{T}q1_{i}^{T}\mathbf{f}_{i+1} \\ pq\mathbf{f}qp=p1_{i}^{T}q1_{i}^{T}\mathbf{f}_{i+1}q1_{i}p1_{i} \end{matrix}$$

As one can see from Eqs. А1.20, А1.24, $\mathbf{c}_{\mathbf{m}}^{-\mathbf{1}}$ is symmetric quasiseparable matrix

$$\left\{\mathbf{c}_{\mathbf{m}}^{-\mathbf{1}}\right\}_{ij}=p5_{i}\mathbf{a2}_{ij}q5_{j},\left\{\mathbf{c}_{\mathbf{m}}^{-\mathbf{1}}\right\}_{ij}=\left\{\mathbf{c}_{\mathbf{m}}^{-\mathbf{1}}\right\}_{ji},i>j,\left\{\mathbf{c}_{\mathbf{m}}^{-\mathbf{1}}\right\}_{ii}=d5_{ii}$$

with components given by recurrent equations

$\begin{matrix} q5=q1 \\ p5_{i}=p2_{i}g1_{i}+q1_{i}^{T}\mathbf{f}_{i+1}-q1_{i}^{T}\mathbf{f}_{i+1}q1_{i}p1_{i} \\ d5_{i}=g1_{i}+q1_{i}^{T}\mathbf{f}_{i+1}q1_{i} \\ p5_{N}=p2_{N}g1_{N} \\ d5_{N}=g1_{N} \end{matrix}$, (А1.25)

where $g1=g^{-1}$.

Calculation of Trace of the Matrix Product

Another useful operation is the operation of the form –$Tr\left(\mathbf{F}\cdot\mathbf{C}\right)$, where $\mathbf{F}$ - symmetric $N_{t}\times N_{t}$ semiseparable or quasiseparable matrix, $\mathbf{C}$ - symmetric semiseparable matrix of the same size with components $\mathbf{A}$, $\mathbf{B}$ and diagonal $\mathbf{d}$.

We first consider semiseparable $\mathbf{F}$. In this case the above operation can be represented as:

$Tr\left(\mathbf{F}\cdot\mathbf{C}\right)=2Tr(\mathbf{H}\cdot\mathbf{B})+\sum_{k=1}^{N_{T}} \mathbf{F}_{kk}d_{k}$ (А1.26)

where $\mathbf{H}$ is $\left(N_{t}-1\right)\times N_{S}$ matrix.

Let prove the equality А1.26. Let

$\mathbf{F}_{ki}=\mathbf{F}_{ik}=p_{k}q_{i}$ (А1.27)

Then

$\begin{matrix} \text{Tr}\left(\mathbf{F}\cdot\mathbf{C}\right)=\sum_{k=1}^{N_{t}} \sum_{i=1}^{N_{t}} \mathbf{F}_{ik}\mathbf{C}^{ik}=\sum_{k=1}^{N_{t}} \mathbf{F}_{kk}d_{k}+\sum_{i=1}^{N_{t}-1} \sum_{k=i+1}^{N_{t}} \mathbf{F}_{ik}\mathbf{C}^{ik}+\sum_{i=2}^{N_{t}} \sum_{k=1}^{i-1} \mathbf{F}_{ik}\mathbf{C}^{ik}= \\ \begin{matrix} & & \end{matrix}=\sum_{k=1}^{N_{t}} \mathbf{F}_{kk}d_{k}+\sum_{i=1}^{N_{t}-1} \sum_{k=i+1}^{N_{t}} \mathbf{F}_{ik}\mathbf{C}^{ik}+\sum_{k=1}^{N_{t}-1} \sum_{i=k+1}^{N_{t}} \mathbf{F}_{ik}\mathbf{C}^{ik}=\sum_{k=1}^{N_{t}} \mathbf{F}_{kk}d_{k}+\sum_{i=1}^{N_{t}-1} \sum_{k=i+1}^{N_{t}} \left(\mathbf{F}_{ik}+\mathbf{F}_{ki}\right)\mathbf{C}^{ik} \\ \\ \text{Tr}\left(\mathbf{F}\cdot\mathbf{C}\right)=\sum_{k=1}^{N_{t}} \mathbf{F}_{kk}d_{k}+2\sum_{k=1}^{N_{t}-1} \sum_{i=k+1}^{N_{t}} \mathbf{F}_{ik}\mathbf{C}^{ik}=\sum_{k=1}^{N_{t}} \mathbf{F}_{kk}d_{k}+2\sum_{k=1}^{N_{t}-1} p_{k}\left(\sum_{i=k+1}^{N_{t}} q_{i}A_{i}\right)B_{k}= \\ \begin{matrix} & & \end{matrix}=\sum_{k=1}^{N_{t}} \mathbf{F}_{kk}d_{k}+2\sum_{k=1}^{N_{t}-1} H_{k}B_{k} \end{matrix}$(А1.28)

Matrix $\mathbf{H}$ can be evaluated recursively:

$\begin{matrix} H_{i}=p_{i}\mathbf{z}^{i}\begin{matrix} , & i<N_{t} \end{matrix} \\ \mathbf{z}^{i}=\mathbf{z}^{i+1}+q_{i+1}A_{i+1}\begin{matrix} , & i<N \end{matrix}_{t} \\ \mathbf{z}^{N_{t}}=0 \end{matrix}$ (А1.29)

Stabilization of these calculations is similar to Eq. А1.14, provided that all eigenvalues of the matrix $\mathbf{F}$ are zero.

$\begin{matrix} H1_{i}=H_{i}\circ\exp\left(-\lambda^{T}t_{i}\right),i<N_{t} \\ \left\{\mathbf{z1}^{i}\right\}_{jk}=\left\{\mathbf{z1}^{i+1}+q_{i+1}A'_{i+1}\right\}_{jk}\exp(-\lambda_{k}\Delta t)\begin{matrix} , & i<N_{t} \end{matrix} \\ \mathbf{z1}^{N_{t}}=0 \\ H1_{i}=p_{i}\mathbf{z1}^{i}\begin{matrix} , & i<N_{t} \end{matrix} \\ Tr\left(\mathbf{F}\cdot\mathbf{C}\right)=\sum_{k=1}^{N_{t}} \mathbf{F}_{kk}d_{k}+2\sum_{k=1}^{N_{t}-1} H1_{k}B1_{k} \end{matrix}$ (А1.30)

Here and $\left\{\mathbf{z1}^{i}\right\}_{jk}=\left\{\mathbf{z}^{i}\right\}_{jk}\exp\left(-\lambda_{k}t_{i}\right)$ or $\mathbf{z1}^{i}=\mathbf{z}^{i}\circ\exp(-\lambda^{T}t_{i})$ and $A'_{i},B'_{i}$ are given by

$\begin{matrix} A_{i}=A'_{i}\circ e^{\lambda^{T}t_{i}} \\ B_{i}=B'_{i}\circ e^{-\lambda t_{i}} \end{matrix}$ (A1.31)

In the case when $\mathbf{F}$ is quasiseparable matrix, by analogy with Eq. А1.28 we have

$Tr\left(\mathbf{F}\cdot\mathbf{C}\right)=\sum_{k=1}^{N_{t}} \mathbf{F}_{kk}d_{k}+2\sum_{k=1}^{N_{t}-1} q_{k}^{T}\left(\sum_{i=k+1}^{N_{t}} \mathbf{a2}_{ik}^{T}p_{i}^{T}A_{i}\right)B_{k}=\sum_{k=1}^{N_{t}} \mathbf{F}_{kk}d_{k}+2\sum_{k=1}^{N_{t}-1} H_{k}B_{k}$ (А1.32)

and matrix $\mathbf{H}$ is again evaluated recursively:

$\begin{matrix} \mathbf{z}^{N_{t}}=0 \\ \mathbf{z}^{k}=\mathbf{a}_{k+1}^{T}\mathbf{z}^{k+1}+p_{k+1}^{T}A_{k+1}\begin{matrix} , & k<N_{t} \end{matrix} \\ H_{k}=q_{k}^{T}\mathbf{z}^{k}\begin{matrix} , & k<N_{t} \end{matrix} \end{matrix}$ (А1.33)

Stabilization of these calculations could be done in the same way as it was done for Eq. А1.30, provided that eigenvalues $\lambda$ are the same for $\mathbf{F}$and $\mathbf{C}$ (this is indeed the case for the matrices that appear in the equations for the log-likelihood gradient):

$\begin{matrix} H1_{j}=H_{j}\circ\exp\left(-\lambda^{T}t_{j}\right) \\ H1_{N}=0 \\ \left\{\mathbf{z1}^{i}\right\}_{jk}=\left\{a'_{i+1}\mathbf{z1}^{i+1}+p'_{i+1}^{T}A_{i+1}\right\}_{jk}\exp(\lambda_{j}\Delta t+\lambda_{k}\Delta t)\begin{matrix} , & i<N \end{matrix} \\ H1_{i}=q'_{i}^{T}\mathbf{z1}^{i}\begin{matrix} , & i>1 \end{matrix} \\ Tr\left(\mathbf{F}\cdot\mathbf{C}\right)=\sum_{k=1}^{N_{t}} \mathbf{F}_{kk}d_{k}+2\sum_{k=1}^{N_{t}-1} H1_{k}B1_{k} \end{matrix}$ (А1.34)

Where$p'_{i},q'_{i}$ $A'_{i},B'_{i}$ are given by

$\begin{matrix} p_{i}=p'_{i}\circ e^{\lambda^{T}t_{i}} \\ q_{i}=q'_{i}\circ e^{-\lambda t_{i}} \\ A_{i}=A'_{i}\circ e^{\lambda^{T}t_{i}} \\ B_{i}=B'_{i}\circ e^{-\lambda t_{i}} \end{matrix}$ (A1.35)

and$\left\{\mathbf{z}^{i}\right\}_{jk}=\left\{\mathbf{z1}^{i}\right\}_{jk}\exp\left(-(\lambda_{j}t_{i}+\lambda_{k}t_{i})\right)$, $\left\{\mathbf{a}_{i}\right\}_{jk}=\left\{\mathbf{a}'_{i}\right\}_{jk}\exp\left(-\lambda_{j}t_{i}+\lambda_{k}t_{i}\right)$

## А2

## Log-likelihood Gradient

Consider Eq. 20 of Methods

$$\nabla\log L=-\frac{1}{2}\sum_{i=1}^{N} \left(-2\left(\nabla\overline{I}\right)^{T}\mathbf{c}_{\mathbf{m}}^{-\mathbf{1}}\left(I_{1:N_{t}}^{i}-\overline{I}\right)-\left(I_{1:N_{t}}^{i}-\overline{I}\right)^{T}\mathbf{c}_{\mathbf{m}}^{-\mathbf{1}}\nabla\mathbf{c}_{\mathbf{m}}\mathbf{c}_{\mathbf{m}}^{-\mathbf{1}}\left(I_{1:N_{t}}^{i}-\overline{I}\right)\right)-\frac{N}{2}\nabla\log\left(\det\left(\mathbf{c}_{\mathbf{m}}\right)\right)$$

(А2.1)

It can be represented in the form

$\nabla\log L=-\frac{1}{2}(F_{1}+F_{2}+F_{3}+F_{4}+F_{5})$ (А2.2)

where $F_{1}$, $F_{2}$,$F_{3}$,$F_{4}$,$F_{5}$ satisfy the following equations (we keep in mind that $\text{Tr}(\mathbf{AB})=\text{Tr}(\mathbf{BA})$, $\text{Tr}(\mathbf{A}^{\mathbf{T}})=\text{Tr}(\mathbf{A})$ and $\log(\det(\mathbf{A}))=\text{Tr}(\log\mathbf{A})$):

$-\sum_{i=1}^{N} \left(\left(I_{1:N_{t}}^{i}-\overline{I}\right)^{T}\mathbf{c}_{m}^{-1}\nabla\mathbf{c}_{\mathbf{m}}\mathbf{c}_{\mathbf{m}}^{-\mathbf{1}}\left(I_{1:N_{t}}^{i}-\overline{I}\right)\right)=-Tr\left(\mathbf{c}_{2}\mathbf{c}_{2}^{T}\nabla\mathbf{c}_{m}^{1}\right)+Tr\left(\mathbf{c}_{2}\mathbf{c}_{2}^{T}\nabla\left(\frac{\overline{I}\left(\overline{I}\right)^{T}}{N_{ch}}\right)\right)=F_{1}+F_{2}$ (А2.3)

$F_{1}=-Tr\left(\mathbf{c}_{\mathbf{2}}\mathbf{c}_{2}^{T}\nabla\mathbf{c}_{\mathbf{m}}^{\mathbf{1}}\right)$ (А2.4)

$\nabla\mathbf{c}_{\mathbf{m}}^{\mathbf{1}}=\nabla\left(\mathbf{c}_{\mathbf{m}}+\frac{\overline{I}\left(\overline{I}\right)^{T}}{N_{ch}}\right)$ (А2.5)

$F_{2}=Tr\left(\mathbf{c}_{2}\mathbf{c}_{2}^{T}\nabla\left(\frac{\overline{I}\left(\overline{I}\right)^{T}}{N_{ch}}\right)\right)=Tr\left(\mathbf{c}_{2}\mathbf{c}_{2}^{T}\frac{\left(\nabla\overline{I}\right)\left(\overline{I}\right)^{T}}{N_{ch}}+\mathbf{c}_{2}\mathbf{c}_{2}^{T}\frac{\left(\overline{I}\right)\left(\nabla\overline{I}\right)^{T}}{N_{ch}}\right)=2\nabla\left(\overline{I}\right)^{T}\cdot\left(\mathbf{c}_{2}\mathbf{c}_{2}^{T}\frac{\overline{I}}{N_{ch}}\right)$ (А2.6)

$c_{2}^{i}=\mathbf{c}_{\mathbf{m}}^{-\mathbf{1}}\left(I_{1:N_{t}}^{i}-\overline{I}\right)$ (А2.7)

$F_{3}=\sum_{i=1}^{N} \left(-2\left(\nabla\overline{I}\right)^{T}\mathbf{c}_{\mathbf{m}}^{-\mathbf{1}}\left(I_{1:N_{t}}^{i}-\overline{I}\right)\right)=-2\left(\nabla\overline{I}\right)^{T}M_{1}$ (А2.8)

$M_{1}=\mathbf{c}_{\mathbf{m}}^{-\mathbf{1}}\sum_{i=1}^{N} \left(I_{1:N_{t}}^{i}-\overline{I}\right)=\sum_{i=1}^{N} c_{2}^{i}$ (А2.9)

$N\nabla\log\left(\det\left(\mathbf{c}_{\mathbf{m}}\right)\right)=NTr\left(\mathbf{c}_{\mathbf{m}}^{-\mathbf{1}}\nabla\mathbf{c}_{\mathbf{m}}\right)=F_{4}+F_{5}$ (А2.10)

$\begin{matrix} F_{4}=NTr\left(\mathbf{c}_{\mathbf{m}}^{-\mathbf{1}}\nabla\mathbf{c}_{\mathbf{m}}^{\mathbf{1}}\right) \\ F_{5}=-2\left(\nabla\overline{I}\right)^{T}\cdot\left(c_{m}^{-1}\frac{N\overline{I}}{N_{ch}}\right) \end{matrix}$ (А2.11)

where in Eq. A2.6 denotes scalar multiplication, $\nabla c_{m}$ is given by Eq. 20 of Methods and $\nabla\mathbf{c}_{\mathbf{m}}^{\mathbf{1}}$ is given by

, $i\geq j$ (А2.12)

$\left\{\nabla\mathbf{c}_{\mathbf{m}}^{\mathbf{1}}\right\}_{ji}=\left\{\nabla\mathbf{c}_{m}^{1}\right\}_{ij}$ , $i\geq j$ (А2.13)

where matrices $\tilde{\mathbf{A}}$ and $\tilde{\mathbf{B}}$are equal to the matrices $\mathbf{A}=\mathbf{p}$and $\mathbf{B}=\mathbf{q}$ from Eqs. 18 of Methods without last $N_{noise}+1$ columns and rows, respectively (see also Eqs. A0.10-13).

The calculation of $F_{1}$ using Eq. А2.4 is reduced to the operation of the form $Tr\left(\mathbf{F}\cdot\mathbf{C}\right)$ with $\mathbf{C}=\nabla\mathbf{c}_{\mathbf{m}}^{\mathbf{1}}$ and $N_{t}\times N_{t}$ symmetric semiseparable $\mathbf{F}=\mathbf{c}_{2}\mathbf{c}_{2}^{T}$. The calculation of $F_{4}$ using Eq. А2.11 is reduced to the same operation but with $N_{t}\times N_{t}$ quasiseparable $\mathbf{F}=\mathbf{c}_{\mathbf{m}}^{-1}$. As we proved earlier, both operations are represented as

and keeping in mind that $\mathbf{C}$ is identically equal to $\nabla\mathbf{c}_{\mathbf{m}}^{\mathbf{1}}$, using A2.12 we write

(А2.14)

where $\mathbf{H}_{B}$ is a $N_{S}\times N_{t}$ matrix and $\mathbf{H}_{A}$ is $N_{t}\times N_{S}$ matrix, $d_{k}=\left\{\nabla\mathbf{c}_{\mathbf{m}}^{\mathbf{1}}\right\}_{kk}$. $\mathbf{H}_{A}$ and $\mathbf{H}_{B}$ can be calculated recursively (A1, Eqs. А1.29, A1.33 and ultimately used stabilized algorithms A1.30, A1.34). It is reasonable to perform calculations using Eq. А1.30 simultaneously with evaluation of $\mathbf{c}_{2}$ (solving the system $c_{2}^{i}=\mathbf{c}_{\mathbf{m}}^{-\mathbf{1}}\left(I_{1:N_{t}}^{i}-\overline{I}\right)=\left(\mathbf{G}^{-1}\right)^{T}C_{1:N_{t}}^{i}$ with the help of the algorithm А1.10, see also Eqs. 9-11 in Methods), because matrices $\mathbf{z}^{i}$ that appear in Eqs. А1.30 and А1.10 coincide in this case.

It will be shown that the components of $\nabla\tilde{A}$ are equal to zero for all parameters instead of the case when the differentiation is done with respect to the components of the vector$\lambda$. This case will be considered separately.

For the other parameters, Eq. А2.4 is reduced to evaluation of the trace of product of $N_{t}\times N_{s}$matrix $\mathbf{H}_{0}$ by the tensor $\nabla\tilde{\mathbf{B}}$ :

(А2.15)

Similarly, $F_{4}$ (Eq. А2.11) is estimated using Eqs. А1.24, А1.25 from A1

(А2.16)

Introducing additional variables:

$\mathbf{H}_{2}=-\mathbf{H}_{0}+N\mathbf{H}_{1}$ (А2.17)

$\mathbf{H}_{3}=\mathbf{c}_{2}(\mathbf{c}_{2}^{T}\frac{\overline{I}}{N_{ch}})-M_{1}-\mathbf{c}_{\mathbf{m}}^{-\mathbf{1}}\frac{N\overline{I}}{N_{ch}}$ (А2.18)

from Eqs. A2.2, A2.15, A2.16, A2.17, A2.18 we have:

(А2.19)

To calculate $\nabla\mathbf{B}$ and $\nabla\overline{I}$ it is convenient to introduce auxiliary variables $a$ and $b$:

$\begin{matrix} a_{oi}=\mathbf{U}\left(o,i\right)\sum_{j=1}^{N_{S}} \mathbf{U}^{-1}\left(i,j\right)p_{j}\left(0\right) \\ b_{o'ok}=\mathbf{U}\left(o,k\right)\mathbf{U}^{-1}\left(k,o'\right) \end{matrix}$ (А2.20)

where $\mathbf{U}$ is the matrix composed of the rate matrix eigenvectors and $p_{j}\left(0\right)$ is initial state probability vector (see A0 and Eqs. 18 in Methods).

Therefore, we have

$p_{o}\left(t\right)=\sum_{i=1}^{N_{S}} e^{\lambda_{i}t}\mathbf{U}\left(o,i\right)\sum_{j=1}^{N_{S}} \mathbf{U}^{-1}\left(i,j\right)p_{j}\left(0\right)=\sum_{i=1}^{N_{S}} e^{\lambda_{i}t}a_{oi}$ (А2.21)

$$\bar{I}(t)=N_{ch}\sum_{o=1}^{N_{O}} i_{o}p_{o}\left(t\right)$$

(A2.22)

Let introduce a new parameter vector $\theta^{N}=\left[N_{ch},i_{o},a,b,\lambda\right]$ instead of the model parameter vector $\theta=\left[k,i,N_{ch}\right]$ (see Methods, The Model of a Macroscopic Current). The log-likelihood gradient with respect to $\theta^{N}$ can be easily computed:

$\frac{\partial}{\partial N_{ch}}$: $\frac{\partial\overline{I\left(t\right)}}{\partial N_{ch}}=\sum_{o=1}^{N_{O}} i_{o}p_{o}\left(t\right)$, , (А2.23)

$\frac{\partial}{\partial i_{o}}$:$\frac{\partial\overline{I\left(t\right)}}{\partial i_{o}}=N_{ch}p_{o}\left(t\right)$,, (А2.24)

$\frac{\partial}{\partial a_{\alpha\beta}}$ : $\frac{\partial\overline{I\left(t\right)}}{\partial a_{\alpha\beta}}=N_{ch}i_{\alpha}e^{\lambda_{\beta}t}$, , (А2.25)

$\frac{\partial}{\partial b_{\alpha\beta\gamma}}$: $\frac{\partial\overline{I\left(t\right)}}{\partial b_{\alpha\beta\gamma}}=0$, , (А2.26)

Thus for all $\theta_{i}^{N}$ except the components of vector $\lambda$ it appears that $\frac{\partial\tilde{\mathbf{A}}}{}\partial\theta_{i}^{N}=0$ and, therefore, in Eq. А2.14 $\mathbf{H}_{\nabla}=0$. In this case log-likelihood gradient is calculated using Eq. А2.19.

Consider the derivatives of log-likelihood with respect to the components of vector $\lambda$:

$\frac{\partial\mathbf{c}_{\mathbf{m}}^{\mathbf{1}}\left(t_{i},t_{j}\right)}{\partial\lambda_{\alpha}}=N_{ch}\sum_{o'=1}^{N_{O}} i_{o'}\left(\sum_{k=1}^{N_{S}} e^{\lambda_{k}\left(t_{i}-t_{j}\right)}\left(t_{j}e^{\lambda_{\alpha}t_{j}}a_{o'\alpha}\right)\sum_{o=1}^{N_{O}} \left(i_{o}b_{o'ok}\right)+\left(t_{i}-t_{j}\right)e^{\lambda_{\alpha}\left(t_{i}-t_{j}\right)}p_{o'}\left(t_{j}\right)\sum_{o=1}^{N_{O}} \left(i_{o}b_{o'o\alpha}\right)\right)=\left\{\mathbf{Q}_{\mathbf{2}}+\mathbf{Q}_{\mathbf{1}}+\mathbf{Q}_{\mathbf{0}}\right\}_{ij}$ (А2.27)

where

where

$\begin{matrix} \left\{\mathbf{Q}_{\mathbf{0}}\right\}_{ij}=N_{ch}\sum_{o'=1}^{N_{O}} i_{o'}t_{i}e^{\lambda_{\alpha}\left(t_{i}-t_{j}\right)}p_{o'}\left(t_{j}\right)\sum_{o=1}^{N_{O}} \left(i_{o}b_{o'o\alpha}\right) \\ \left\{\mathbf{Q}_{\mathbf{1}}\right\}_{ij}=-N_{ch}\sum_{o'=1}^{N_{O}} i_{o'}t_{j}e^{\lambda_{\alpha}\left(t_{i}-t_{j}\right)}p_{o'}\left(t_{j}\right)\sum_{o=1}^{N_{O}} \left(i_{o}b_{o'o\alpha}\right) \\ \left\{\mathbf{Q}_{\mathbf{2}}\right\}_{ij}=N_{ch}\sum_{o'=1}^{N_{O}} i_{o'}\sum_{k=1}^{N_{S}} e^{\lambda_{k}\left(t_{i}-t_{j}\right)}\left(t_{j}e^{\lambda_{\alpha}t_{j}}a_{o'\alpha}\right)\sum_{o=1}^{N_{O}} \left(i_{o}b_{o'ok}\right) \end{matrix}$ (А2.28)

Since $F_{1}+F_{4}=\sum_{i=0}^{2} Tr\left(\mathbf{F}\cdot\mathbf{Q}_{\mathbf{i}}\right)$, where $\mathbf{F}=-\mathbf{c}_{2}\mathbf{c}_{2}^{T}+N\mathbf{c}_{\mathbf{m}}^{-\mathbf{1}}$, $i\in\{0,1,2\}$, then consider, one by another,$\Phi_{i}=Tr\left(\mathbf{F}\cdot\mathbf{Q}_{\mathbf{i}}\right)$.

1) According to Eqs. А1.28, А1.32, А2.15, А2.16 $\Phi_{0}$ can be represented as

(А2.29)

Where and $\mathbf{p}5$, $\mathbf{q}5$, $\mathbf{a}2$- components of the matrix $\mathbf{c}_{\mathbf{m}}^{-\mathbf{1}}$

Matrix $\mathbf{H}_{4}$can be calculated recursively (using Eqs. А1.29, А1.33, replacing$A$ by $\tilde{A}t$):

$\mathbf{H}_{4}=\mathbf{H}'_{4}+\mathbf{H}''_{4}$ (А2.30)

(А2.31)

(А2.32)

Stabilization of these calculations is done in the same way as it was done for Eqs. А1.30, A1.34

2) $\Phi_{1}$ is estimated by Eqs. А2.15, А2.16:

$\text{Φ}_{1}=\sum_{k=1}^{N_{t}} \left(-\sum_{i=1}^{N} \left(c_{2,k}^{i}\right)^{2}+\{\mathbf{c}_{\mathbf{m}}^{-\mathbf{1}}\}_{kk}\right)d_{k}^{1}+2\sum_{k=1}^{N_{t}-1} H_{2,k}B_{k}^{1}$ (А2.33)

where $B_{\alpha,t}^{1}=$ $-N_{ch}\sum_{o'=1}^{N_{O}} i_{o'}te^{-\lambda_{\alpha}t}p_{o'}\left(t\right)\sum_{o=1}^{N_{O}} \left(i_{o}b_{o'o\alpha}\right)$

3) $\Phi{}_{2}$ can be represented as

$\Phi_{2,\alpha}=$ $\sum_{k=1}^{N_{t}} \left(-\sum_{i=1}^{N} \left(c_{2,k}^{i}\right)^{2}+\{\mathbf{c}_{\mathbf{m}}^{-\mathbf{1}}\}_{kk}\right)d_{k}^{2}+2N_{ch}\sum_{t=1}^{N_{t}-1} \sum_{o'=1}^{N_{O}} i_{o'}\sum_{k=1}^{N_{S}} H_{2,k,t}\left(te^{\lambda_{\alpha}t}a_{o'\alpha}\right)\sum_{o=1}^{N_{O}} \left(i_{o}b_{o'ok}\right)$ (А2.34)

This results in the expression for log-likelihood gradient with respect to the components of the vector $\lambda$:

(А2.35)

where:

(A2.36)

To find the log-likelihood gradient with respect to the parameters $\theta$ (instead of $\theta^{N}$) in this work we make use of equation

$\frac{\partial\log L}{\partial\theta_{j}}=\sum_{i=1}^{N_{\theta^{N}}} \frac{\partial\log L}{\partial\theta_{i}^{N}}\frac{\partial\theta_{i}^{N}}{\partial\theta_{j}}$ (А2.37)

where $\frac{\partial\theta_{i}^{N}}{\partial\theta_{j}}$ was estimated as follows:

$\frac{\partial\theta_{i}^{N}}{\partial\theta_{j}}\approx\frac{\theta_{i}^{N}\left(\theta_{1},...\theta_{j}+\Delta\theta,...\right)-\theta_{i}^{N}\left(\theta\right)}{\Delta\theta}$ (А2.38)

Let us estimate the number of elementary operations required for estimation of the log-likelihood gradient. Evaluation of all matrices $\mathbf{H}_{i}$, $i\in\{1,2,3,4\}$ using Eqs. А1.29, А1.33, А2.30-32 requires approximately $4N_{S}N_{T}N+2N_{S}^{2}N_{T}$ operations. The size of the vector $\theta^{N}$ is $\left(N_{O}^{2}+N_{O}+1\right)N_{S}+N_{O}+1$. Evaluation of the log-likelihood gradient with respect to variables $b$ requires about $(N_{O}+1)N_{S}N_{T}$ operations, with respect to $a$ - $(N_{O}+3)N_{S}N_{T}$, with respect to $i$ - $3(N_{S}+1)N_{O}N_{T}$, with respect to $\lambda$ - $\left(3(N_{O}+1)N_{S}+2N_{O}+1\right)N_{T}$ and with respect to$N_{ch}$ - $(N_{S}+1)N_{T}$ operations.

Calculation of $\frac{\partial\theta_{i}^{N}}{\partial\theta_{j}}$ with Eq. А2.38 requires ~$N_{S}^{3}N_{\theta}$ operations. Thus, for models with relatively small number of open states, which means that $N_{O}$ is not very large ($N_{O}<<\frac{N}{2}$), the time for computing matrices $\mathbf{H}_{i}$ will mainly limit the log-likelihood gradient computation time.

Summing up, the log-likelihood gradient computation is approximately 2 times slower then the computation of the log-likelihood itself.

## А3

## Log-likelihood Calculation in the Case of Complex Protocol

Since in the case of complex protocol covariance matrix satisfies the equation

$\begin{matrix} \left\{\mathbf{c}_{\mathbf{m}}\right\}_{ij}=\sum_{k=1}^{N_{S}+1} \mathbf{A}_{ik}\left(\prod_{\nu=s\left(j\right)+1}^{s(i)-1} e^{\mathbf{K}'_{\nu}(T_{\nu}-T_{\nu-1})}\right)\mathbf{B}_{kj}\begin{matrix} , & i\geq j \end{matrix} \\ \left\{\mathbf{c}_{\mathbf{m}}\right\}_{ji}=\left\{\mathbf{c}_{\mathbf{m}}\right\}_{ij}\begin{matrix} , & i\geq j \end{matrix} \end{matrix}$ (А3.1)

(Eqs. A0.10-13), then it is quasiseparable (see А1.19), i.e. its elements can be represented as

$\begin{matrix} \{\mathbf{c}_{\mathbf{m}}\}_{ij}=p_{i}\mathbf{a}_{i-1}\mathbf{a}_{i-2}...\mathbf{a}_{j+1}q_{j}\begin{matrix} , & i>j \end{matrix} \\ \{\mathbf{c}_{\mathbf{m}}\}_{ji}=\{\mathbf{c}_{\mathbf{m}}\}_{ij}\begin{matrix} , & i>j \end{matrix} \\ \{\mathbf{c}_{\mathbf{m}}\}_{ii}=d_{i} \end{matrix}$ (А3.2)

where $\mathbf{a}_{i}=\mathbf{E}$ for $i\notin\left\{s_{1},s_{2},...s_{N_{stim}}\right\}$, $\mathbf{a}_{i}=e^{\mathbf{K'}_{i}(T_{i}-T_{i-1})}$ for $i\in\left\{s_{1},s_{2},...s_{N_{stim}}\right\}$, where $\mathbf{E}$is identity matrix. To solve the problem of representation of the components of matrices $\mathbf{p}$ and $\mathbf{q}$via floating–point numbers it is convenient to use matrices $\mathbf{p}'$ and $\mathbf{q}'$, such that:

$$p'_{i}=p_{i}\circ\exp\left(-\lambda\left\{\frac{t_{i}}{\Delta T}\right\}\Delta T\right)$$

$$q'_{i}=q_{i}\circ\exp\left(\lambda\left\{\frac{t_{i}}{\Delta T}\right\}\Delta T\right)$$

instead of matrices $\mathbf{p}$and $\mathbf{q}$. In this expressions $\left\{x\right\}$ mean integer part of $x$and $\Delta T$ should be selected such that all elements of $\mathbf{p}'$ and $\mathbf{q}'$would be bounded by the limits of floating-point number representation.

It is easy to make sure that $c_{m}$ can be represented as:

$\begin{matrix} \{\mathbf{c}_{\mathbf{m}}\}_{ij}=p'_{i}\mathbf{a}_{i-1}\mathbf{a}_{i-2}...\mathbf{a}_{j+1}q'_{j}\begin{matrix} , & i>j \end{matrix} \\ \{\mathbf{c}_{\mathbf{m}}\}_{ji}=\{\mathbf{c}_{\mathbf{m}}\}_{ij}\begin{matrix} , & i>j \end{matrix} \\ \{\mathbf{c}_{\mathbf{m}}\}_{ii}=d_{i} \end{matrix}$ (А3.3)

where $\mathbf{a}_{i}=diag\left(\exp\left(\lambda\Delta T\right)\right)$ for $i$ such that $\mathrm{mod}(t_{i},\Delta T)=0$, $\mathbf{a}_{i}=e^{\mathbf{K'}_{i}(T_{i}-T_{i-1})}$ for $i\in\left\{s_{1},s_{2},...s_{N_{stim}}\right\}$ and $\mathbf{a}_{i}=\mathbf{E}$ for all others $i$. Analogous scheme of calculations is applicable for the case of simple protocol.

Cholesky decomposition of quasiseparable $\mathbf{c}_{\mathbf{m}}$ matrix (А1.1) is done similar to that of semiseparable $\mathbf{c}_{\mathbf{m}}$ (Eqs. А1.2-А1.5):

$\begin{matrix} \left\{\mathbf{c}_{m}\right\}_{ij}=\sum_{k=1}^{j} \mathbf{G}_{ik}\mathbf{G}_{jk}g_{k}=\sum_{k=1}^{j-1} p1_{i}\mathbf{a2}_{ik}q1_{k}p1_{j}\mathbf{a2}_{jk}q1_{k}g_{k}+g_{j}p1_{i}\mathbf{a2}_{ij}q1_{j}\begin{matrix} , & i>j \end{matrix} \\ \left\{\mathbf{c}_{m}\right\}_{ij}=p1_{i}\mathbf{a2}_{ij-1}\sum_{k=1}^{j-1} \mathbf{a2}_{jk}q1_{k}p1_{j}\mathbf{a2}_{jk}q1_{k}g_{k}+g_{j}p1_{i}\mathbf{a2}_{ij}q1_{j}=p1_{i}\mathbf{a}2_{ij}\left(\mathbf{a}_{j}\left(\sum_{k=1}^{j-1} \mathbf{a2}_{jk}q1_{k}\left(\mathbf{a2}_{jk}q1_{k}\right)^{T}g_{k}\right)p1_{j}^{T}+g_{j}q1_{j}\right) \\ d_{j}=p1_{j}\left(\sum_{k=1}^{j-1} \mathbf{a2}_{jk}q1_{k}\left(\mathbf{a2}_{jk}q1_{k}\right)^{T}g_{k}\right)p1_{j}^{T}+g_{j}\begin{matrix} , & j>1 \end{matrix} \\ g_{1}=d_{1} \end{matrix}$ (А3.4)

Therefore,

$\begin{matrix} p1=p \\ q_{j}=\mathbf{a}_{j}\left(\sum_{k=1}^{j-1} \mathbf{a2}_{jk}q1_{k}\left(\mathbf{a2}_{jk}q1_{k}\right)^{T}g_{k}\right)p1_{j}^{T}+g_{j}q1_{j} \end{matrix}$ (А3.5)

matrix $\mathbf{f}_{j}=\left(\sum_{k=1}^{j-1} \mathbf{a2}_{jk}q1_{k}\left(\mathbf{a2}_{jk}q1_{k}\right)^{T}g_{k}\right)$ satisfies recurrent equation

$\mathbf{f}_{j}=\mathbf{a}_{j-1}\mathbf{f}_{j-1}\mathbf{a}_{j-1}^{T}+q1_{j-1}q1_{j-1}^{T}g_{j-1}$ (А3.6)

Similar recurrent relations hold for $g$ and $q1$

$\begin{matrix} g_{j}=d_{j}-p_{j}\mathbf{f}_{j}p1_{j}^{T} \\ q1_{j}=\frac{q_{j}-\mathbf{a}_{j}\mathbf{f}_{j}p1_{j}^{T}}{g_{j}} \end{matrix}$ (А3.7)

For$i\notin\left\{s_{1},s_{2},...s_{N_{stim}}\right\}$ these recurrent relations are equivalent to relations А1.4-1.5

The system of equation А1.6 with matrix $\mathbf{G}$ being quasiseparable is reduced to a system of recurrent relations

$\begin{matrix} y_{i}=\sum_{j=1}^{N_{x}} \mathbf{G}_{ij}x_{j}=\left(\sum_{j=1}^{i-1} p1_{i}\mathbf{a2}_{ij}q1_{j}x_{j}\right)+\mathbf{G}_{ii}x_{i}=p1_{i}\mathbf{z}^{i-1}+x_{i} \\ \mathbf{z}^{i}=\sum_{j=1}^{i} \mathbf{a2}_{ij}q1_{j}x_{j}=\mathbf{a}_{i-1}\mathbf{z}^{i-1}+q1_{i}x_{i} \end{matrix}$ (А3.8)

$\begin{matrix} x_{i}=y_{i}-p1_{i}\mathbf{z}^{i-1}\begin{matrix} , & i>1 \end{matrix} \\ x_{1}=y_{1} \end{matrix}$ (A3.9)

These relations are equivalent to А1.7-А1.8 for $i\notin\left\{s_{1},s_{2},...s_{N_{stim}}\right\}$.

Algorithms А3.6-7 and А3.8-9 are used when calculating log-likelihood using Eqs. 9-11 of Methods.

Calculations of the likelihood logarithm gradient can be done similarly to the case of simple stimulation protocol.

Indeed after differentiation of Eq. A3.1 we obtain:

$\begin{matrix} \left\{\nabla\mathbf{c}_{\mathbf{m}}\right\}_{ij}=\sum_{k=1}^{N_{S}+1} \nabla\mathbf{A}_{ik}\left(\prod_{\nu=s\left(j\right)+1}^{s(i)-1} e^{\mathbf{K}'_{\nu}(T_{\nu}-T_{\nu-1})}\right)\mathbf{B}_{kj}+\sum_{k=1}^{N_{S}+1} \mathbf{A}_{ik}\left(\prod_{\nu=s\left(j\right)+1}^{s(i)-1} e^{\mathbf{K}'_{\nu}(T_{\nu}-T_{\nu-1})}\right)\nabla\mathbf{B}_{kj}+\sum_{k=1}^{N_{S}+1} \mathbf{A}_{ik}\nabla\left(\prod_{\nu=s\left(j\right)+1}^{s(i)-1} e^{\mathbf{K}'_{\nu}(T_{\nu}-T_{\nu-1})}\right)\mathbf{B}_{kj}\begin{matrix} , & i\geq j \end{matrix} \\ \left\{\nabla\mathbf{c}_{\mathbf{m}}\right\}_{ji}=\left\{\nabla\mathbf{c}_{\mathbf{m}}\right\}_{ij}\begin{matrix} , & i\geq j \end{matrix} \end{matrix}$ (A3.10)

In the case when $\mathbf{F}$ and $\mathbf{C}$ are quasiseparable matrices, by analogy with Eq. А1.23 we have

(А3.11)

and matrix $\mathbf{H}$ is again evaluated recursively:

(А3.12)

Stabilization of these calculations could be done in the same way as it was done for Eq. А1.30. Thus, similarly to Eq. A2.14 we have

(A3.13)

where $H_{\nabla}$is a tensor of rank 3 which appeared as result of application of the Eq. A3.11-A3.12 to the last term of Eq. 3.10. Finally, similarly to Eq. 2.19 we have:

(A3.14)

Then calculation of $\frac{\partial\log L}{\partial\theta_{s}}$ for all terms of Eq 3.14 except the last term can be done similarly to the calculations in the case of simple protocol as it is described in A2, and they take approximately the same number of elementary operations. To efficiently calculate last term in Eq. A3.14 differentiation should be done with respect to the elements of matrix $a_{s}$ for each time moment $t_{s}$ when stimulation changes. Then using Eq. A3.11 the last term in Eq. A3.14 can be represented as a sum of two terms of the form (these terms match the terms of Eq. А2.17 ):

(A3.15)

Calculation of the last term of Eq. A3.14 using Eq. A3.15 requires an order of

$N_{t}\left(N_{S}N+2N_{S}^{2}\right)N_{stim}+N_{s}^{3}N_{stim}^{2}$ elementary operations (without accounting for $z^{s}$calculation and performing the calculations in the order, marked by parentheses). If $N_{t},N>>N_{s},N_{stim}$, then the maximal number (it depends on the exact form of the complex stimulation protocol) of operations required for the gradient computation is $N_{\nabla\log L}\approx N_{\log L}(2+N_{stim}/2)+N_{s}^{3}N_{stim}^{2}$. Thus the complexity of the $\nabla\log L$ estimation additionally increases approximately on $N_{\log L}N_{stim}/2$ operations compared to the complexity of $\nabla\log L$ computation in the case of the simple protocol.

## А4

## Method Implementation

Algorithms for the estimation of the log-likelihood and its gradient and the macroscopic current generator, described in this work, were implemented in MATLAB (except the algorithm for the log-likelihood gradient calculation in the case of complex protocol of stimulation (A3), described by Eqs.A3.10-3.15. Currently, in the case of complex protocol the log-likelihood gradient has been estimated as a sum of log-likelihood gradients for simple protocols, which are the components of a complex protocol). Source codes are freely available at: <http://code.google.com/p/multi-channel-data-analysis/>.

## A5

## Comparison of Different Algorithms for the Log-likelihood Evaluation

To compare the efficiency of our and Kalman filter based methods for the log-likelihood evaluation, we conducted a series of computational experiments. A set of 200 macroscopic currents, 2000 points each, was generated using the brief stimulation protocol and the standard 7-state GABA_A_ receptor model [30]). The log-likelihood of this set of currents was evaluated using 3 different algorithms: Kalman filter based algorithm implemented in MATLAB, Kalman filter based algorithm implemented in C and our algorithm implemented in MATLAB. Models of larger sizes, taken for the log-likelihood estimation, were obtained by concatenation of the 7-state model [30] with a random diagonal matrices of different sizes.

Time (s), required for one evaluation of the log-likelihood of 200 macroscopic currents 2000 points each depending on the number of states in the ion channel model.

| Model  size  Algorithm | 7 | 10 | 15 | 20 | 30 | 40 | 50 | 60 |
| --- | --- | --- | --- | --- | --- | --- | --- | --- |
| MATLAB, Kalman filter ^*^ | 6.1 | 6.7 | 9.5 | 11 | 17 | 26 | 40 | 57 |
| C, Kalman filter ^**^ | 1.57 | 1.67 | 3.16 | 3.85 | 7 | 12 | 19 | 29 |
| MATLAB,  Semiseparable  representation  of the covariance matrix | 0.082 | 0.089 | 0.101 | 0.121 | 0.142 | 0.179 | 0.220 | 0.263 |

^*^  - Custom implementation of the Kalman filter based recursive algorithm [22].

^**^ - State Space Models Toolbox for MATLAB (http://ssmodels.sourceforge.net/) was used for the implementation of the Kalman filter based algorithm [22].This open source software created by Jyh-Ying Peng supports model construction with MATLAB objects and efficient Kalman filter backend implemented in C.
